# Feasibility of deep learning-based tumor segmentation for target delineation and response assessment in grade-4 glioma using multi-parametric MRI

**DOI:** 10.1093/noajnl/vdad037

**Published:** 2023-04-13

**Authors:** Marianne H Hannisdal, Dorota Goplen, Saruar Alam, Judit Haasz, Leif Oltedal, Mohummad A Rahman, Cecilie Brekke Rygh, Stein Atle Lie, Arvid Lundervold, Martha Chekenya

**Affiliations:** Department of Oncology, Haukeland University Hospital, BergenNorway; University of Bergen, Bergen, Norway; Department of Oncology, Haukeland University Hospital, BergenNorway; University of Bergen, Bergen, Norway; Department of Radiology, Mohn Medical Imaging and Visualization Centre, Haukeland University Hospital, Bergen, Norway; Department of Biomedicine; University of Bergen, Bergen, Norway; Department of Radiology, Haukeland University Hospital, Bergen, Norway; University of Bergen, Bergen, Norway; Department of Radiology, Haukeland University Hospital, Bergen, Norway; Department of Radiology, Mohn Medical Imaging and Visualization Centre, Haukeland University Hospital, Bergen, Norway; University of Bergen, Bergen, Norway; Department of Oncology, Haukeland University Hospital, BergenNorway; Department of Biomedicine; University of Bergen, Bergen, Norway; Department of Radiology, Haukeland University Hospital, Bergen, Norway; University of Bergen, Bergen, Norway; Department of Clinical Odontology; University of Bergen, Bergen, Norway; Department of Radiology, Mohn Medical Imaging and Visualization Centre, Haukeland University Hospital, Bergen, Norway; Department of Biomedicine; University of Bergen, Bergen, Norway; Department of Biomedicine; University of Bergen, Bergen, Norway

**Keywords:** artificial intelligence, glioblastoma, neural networks, radiation therapy, tumor segmentation

## Abstract

**Background:**

Tumor burden assessment is essential for radiation therapy (RT), treatment response evaluation, and clinical decision-making. However, manual tumor delineation remains laborious and challenging due to radiological complexity. The objective of this study was to investigate the feasibility of the HD-GLIO tool, an ensemble of pre-trained deep learning models based on the nnUNet-algorithm, for tumor segmentation, response prediction, and its potential for clinical deployment.

**Methods:**

We analyzed the predicted contrast-enhanced (CE) and non-enhancing (NE) HD-GLIO output in 49 multi-parametric MRI examinations from 23 grade-4 glioma patients. The volumes were retrospectively compared to corresponding manual delineations by 2 independent operators, before prospectively testing the feasibility of clinical deployment of HD-GLIO-output to a RT setting.

**Results:**

For CE, median Dice scores were 0.81 (95% CI 0.71–0.83) and 0.82 (95% CI 0.74–0.84) for operator-1 and operator-2, respectively. For NE, median Dice scores were 0.65 (95% CI 0.56–0,69) and 0.63 (95% CI 0.57–0.67), respectively. Comparing volume sizes, we found excellent intra-class correlation coefficients of 0.90 (*P* < .001) and 0.95 (*P* < .001), for CE, respectively, and 0.97 (*P* < .001) and 0.90 (*P* < .001), for NE, respectively. Moreover, there was a strong correlation between response assessment in *Neuro-Oncology* volumes and HD-GLIO-volumes (*P* < .001, Spearman’s R^2^ = 0.83). Longitudinal growth relations between CE- and NE-volumes distinguished patients by clinical response: Pearson correlations of CE- and NE-volumes were 0.55 (*P* = .04) for responders, 0.91 (*P* > .01) for non-responders, and 0.80 (*P* = .05) for intermediate/mixed responders.

**Conclusions:**

HD-GLIO was feasible for RT target delineation and MRI tumor volume assessment. CE/NE tumor-compartment growth correlation showed potential to predict clinical response to treatment.

**Key Points**

- HD-GLIO has high geometrical similarity and reliability with manual 3D delineations.
- HD-GLIO predicts both contrast-enhancing (CE)- and non-enhancing (NE) volumes.
- Longitudinal change in CE/NE distinguishes groups of differing treatment responses.

**Importance of Study**

High-precision tumor delineation is a prerequisite for optimal radiation treatment planning that enables precise organ-at-risk sparing and reduction of adverse effects. Furthermore, tumor volume underlines RANO scores and treatment response assessment. However, tumor delineation is both complex and time-consuming, and as a result, single-slice 2D measures often serve as a surrogate for full 3-dimensional (3D) volume measures. We have shown herein that HD-GLIO holds high geometrical similarity and reliability with manual 3D delineations. Since the tool could segment both the contrast-enhancing (CE)- and the non-enhancing (NE) tumor compartment, their relational change in growth pattern became assessable. CE/NE tumor-compartment growth correlation distinguished patients with different treatment responses. The ability of the HD-GLIO tool to differentiate grade-4 glioma tumor compartments may render it an efficient tool for longitudinal assessment of treatment response. Improved quantitative information should allow for better treatment planning, and rapid determination of radiological treatment response.

Glioblastoma (GBM) is the most common and aggressive primary brain tumor in adults, and age-adjusted incidence is approximately 3.2 per 100 000 population.**^1^** The primary treatment for GBM is maximum safe surgical resection, and conformal external beam radiotherapy (RT) with concurrent Temozolomide chemotherapy.^2^ Patients’ prognosis and responses to treatment differ depending on age at diagnosis, Karnofsky Performance Score,^3^ extents of tumor resection, and promoter methylation status of the DNA repair gene O^6^-methylguanine-DNA methyltransferase (MGMT).^4^ Tumors with mutated isocitrate dehydrogenase (IDH) gene that were earlier denoted secondary GBM,^5^ have recently been reclassified as astrocytoma grade IV.^6^ Treatment efficacy is measured by overall survival (OS) and radiologic response in terms of progression-free survival.^7^ We previously reported that recurrent glioblastoma patients harboring unmethylated *MGMT* promoter segregated a priori into three groups with divergent clinical responses^8^ to a chemo-sensitization regimen of bortezomib (BTZ) 48 hours prior to temozolomide (TMZ) treatment. Radiological response is an important metric in evaluating treatment benefits. However, there is a knowledge gap regarding earliest radiological signs of clinical responses during longitudinal follow-up of glioblastoma patients undergoing intervention trials.

In radiation oncology, the target volume delineation remains a time-consuming manual task, where leading guidelines differ in how the gross target volume (GTV) and the clinical target volume (CTV) are defined. According to the European Society for Radiotherapy and Oncology Advisory Committee on Radiation Oncology Practice (ESTRO-ACROP), GTV should include all contrast-enhancing areas on T1-weighted magnetic resonance imaging (MRI), excluding post-surgical infarction or gliosis.^9^ Delineation should not be based on postoperative MRI alone, preoperative scans should also be assessed, providing a more precise GTV. Moreover, CTV is defined by an isotropic margin of 20 mm from the GTV adjusted at anatomical barriers, based upon evidence of infiltration- and recurrence patterns.^9^ However, the Radiation Therapy Oncology Group (RTOG) advises inclusion of peritumoral edema in the GTV since it is thought to hold high concentrations of non-enhancing tumor cells.^10^ RTOG also advises additional CTV margin of 20 mm^10^ (Supplementary Figure 1). However, tumors delineated by either guideline do not differ in recurrence pattern or patient survival.^11^ The consequence of poorly demarcated target volume is damage to healthy brain tissue, inflicting increased radionecrosis and demyelinization with potential long-term neurocognitive deficits.^11^ Suboptimal target coverage may lead to reduced therapeutic effects. Manual target delineation is arguably the weakest link throughout the course of the RT planning process.^12,13^ Profound inter-center variability in GBM RT target delineation practice between the European treatment institutions has been reported.^14^ Hence, there is a need for improved methods that reduce incongruity in tumor volume evaluation between professionals and alleviate workload.

Quantitative tumor burden assessment in *Neuro-Oncology* radiology is important as objective treatment response and progression-free survival are considered reliable endpoints. The response assessment in neuro-oncology (RANO) working group criteria is the current standard.^15^ The clinical response assessment in RANO is based on 2 perpendicular measures of the largest part of the contrast-enhancing lesion, following the given criteria of volumetric duration, evaluated in relation to the use of corticosteroids, new lesions, and clinical status. Several studies have compared the use of 2 perpendicular measures, to 3D-volumetric segments,^16–19^ where consensus states the 3D-approach supersedes 2-dimensional in both accuracy and reliability. Nevertheless, in clinical practice, there has been a compromise that practical efficiency outweighs the inaccuracy of 2-dimensional measures. This underscores the unmet need for more effective tools for quantifying true volumetric tumor burden.

Recently, a deep learning (DL) brain tumor segmentation tool, HD-GLIO, was developed at the Heidelberg University Hospital (UKHD)/ German Cancer Research Center (DKFZ). The tool comprises an ensemble of DL models based on the nnUNet algorithm^17^ and trained by neuro-radiologist’s tumor labeling to automatically segment (1) the T1 contrast-enhancing (CE) volume, and (2) the non-enhancing (NE) volume corresponding to T2-FLAIR hyperintense abnormality.^17,20^ In this study, we compared the prediction of the HD-GLIO tool to the oncologist’s and neuroradiologists manual delineation outputs, clinical RANO measures, as well as longitudinal tumor compartment development to clinical treatment response. Additionally, we tested prospectively the feasibility of clinical deployment of HD-GLIO output in radiation treatment planning for a limited group of patients.

## Aim of the Study

We hypothesized that segmenting glioblastoma and grade-4 astrocytoma tumor compartments on multi-parametric MRI (mpMRI) using HD-GLIO will be geometrically similar to manual delineations of tumors from patients undergoing treatment in the phase 1B/II BORTEM-17 (ClinicalTrials.gov Identifier: NCT03643549). Furthermore, we aimed to investigate the feasibility of clinical deployment of this DL tool as an oncologist support tool for RT target delineation, and volumetric MRI tumor burden assessment. Additionally, we aimed to identify any unique features in longitudinal MRI data that may be correlated to treatment response.

## Materials and Methods

### Data Material

The study was approved by the Regional Ethics Committee in Western Norway, reference number 2017/2084/REK vest and Norwegian Medicines Agency (17/17445-17). All eligible patients signed the approved consent form for study participation. We analyzed 49 mpMRI datasets, 29 retrospectively, and 20 prospectively.

The 29 retrospective datasets were from 13 patients harboring recurrent grade-4 gliomas, comprising 10 glioblastoma patients and 3 grade-4 astrocytoma patients with unmethylated MGMT promoter, included in the ongoing multicentre study (ClinicalTrials.gov Identifier: NCT03643549). These participants were selected according to the following inclusion criteria: (1) histologically and radiologically confirmed recurrent or progressed WHO-grade-4^21^ intracranial glioma, (2) ≥ 12 weeks since radiation treatment, (3) estimated glomerular filtration rate ≥ 60, (4) negative pregnancy test, and, (5) age > 18. See ClinicalTrials.gov Identifier: NCT03643549 for complete list of inclusion/exclusion criteria. For these 13 patients, image acquisition was performed at 3T Siemens MAGNETOM Prisma (Siemens Healthineers, Munich, Germany) MR scanner at Haukeland University Hospital (HUH).

Furthermore, 10 patients harboring grade-4 primary gliomas, comprising 7 glioblastoma patients and 3 grade-4 astrocytoma patients, were included for prospective validation using 20 clinical pre- and postoperative mpMRI datasets. Image acquisition was performed on MR scanners within HUH (field strength 1.5–3T), according to clinical routine. Inclusion criteria for prospective patients were: (1) newly diagnosed, histologically confirmed WHO-grade-4 intracranial glioma,^6^ (2) available standard mpMRI, and (3) were to receive conformal RT during the period of November 2022–March 2023. Exclusion criteria included previous RT treatment.

All de-identified mpMRI datasets (*n* = 49) were stored on the Secure Access to Research Data and E-Infracture (SAFE) facility at the University of Bergen, analyzed with the HD-GLIO tool at the Mohn Medical Imaging and Visualization Center, Department of Radiology at HUH. The number of included patients concurred with ESTRO recommendations for implementing artificial intelligence-based applications in radiotherapy.^22^

### Multi-parametric MRI and HD-GLIO Model

The 13 patients harboring recurrent tumors underwent baseline MRI examinations (session-1) followed by mpMRI every 56 days (session-2, …→) up to 4 sessions, parallel to sequential combination of bortezomib (BTZ) 48 hours before temozolomide (TMZ) 150–200 mg/m2 treatment in 5-day cycles.^8^ The MRI sequences used in this study included 3D T1-weighted pre- (T1-w) and post-contrast (cT1-w), axial T2-weighted (T2-w), and 3D T2 fluid-attenuated inversion recovery (T2-FLAIR) recordings. The MRI protocol is in line with the consensus recommendations for standardized brain tumor imaging protocol in clinical trials,^23^ and corresponds to the mpMRI protocol used to train HD-GLIO.

The HD-GLIO tool was selected because it is a pre-trained open-sourced ensemble model, that is well documented,^17,20,24^ readily available and maintained at the Division of Computational Neuroimaging, Heidelberg University Hospital and Division of Medical Image Computing, German Cancer Research Center code-hosting GitHub repository (https://github.com/NeuroAI-HD/HD-GLIO). Moreover, the HD-GLIO requirements regarding image acquisition and interpretation are largely in consensus with the protocol in use at HUH, being a clinical proton therapy patient-exchange collaborator. HD-GLIO prediction-output image files were imported into the clinical RT treatment planning system (Eclipse Aria Oncology Information Systems [Varian, California, USA]). The pre-trained HD-GLIO tool used for inference, was originally trained and validated on a large 3220 image dataset consisting of both primary and recurrent GBM from 38 institutions in Europe, and has previously shown good segmentation and 3D tumor volume results.^17^

### Tumor Delineation, Retrospective Analysis

**Figure 1.**
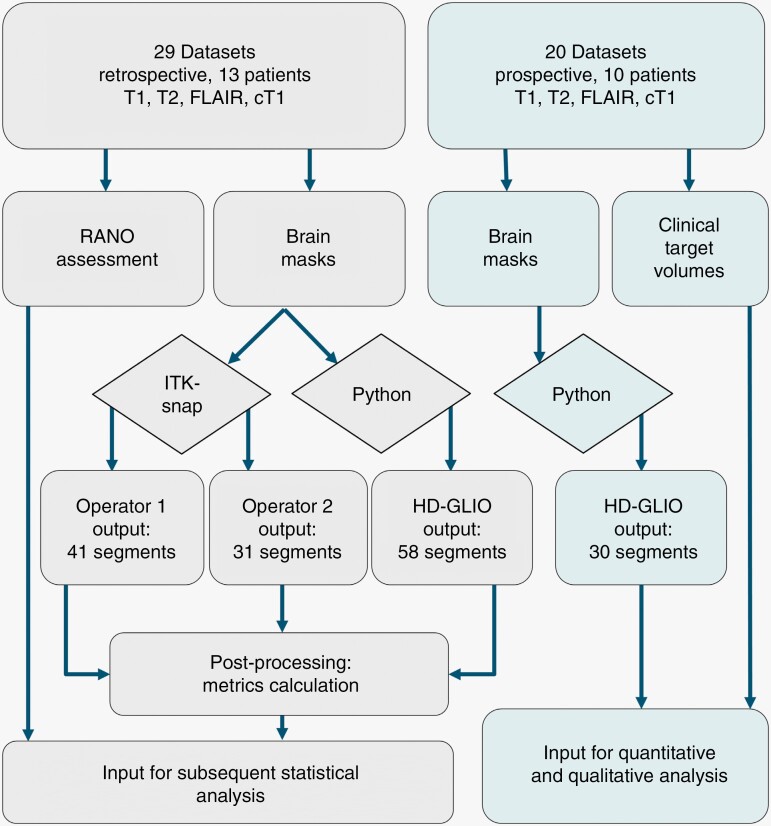
Methodological flowchart. Flowchart showing the methodological steps of the data-processing pipeline for retrospective tumor burden analysis (left) and prospective clinical validation (right).

Two expert clinicians, a consultant oncologist (operator-1) and a consultant neuro-radiologist (operator-2) performed manual 3D delineations independently on the 29 mpMRI datasets, retrospectively, using ITK-snap^25^ version 3.8.0. The operators were selected to reflect the oncologists who delineate the target volume for RT in Scandinavia, and the neuro-radiologists who report the radiological responses on MRI during treatment, where the inter-operator variability addresses the variability across 2 clinical disciplines. The same delineation guidelines as used for training HD-GLIO^17^ were followed. CE region was defined as the contrast-enhancing tumor, excluding necrosis. NE region was defined as T2-FLAIR hyperintense abnormality excluding the contrast-enhancing and necrotic tumor, resection cavity, and obvious leukoaraiosis. In total, 72 manual segmentations were acquired for which 56 were available for inter-operator variability evaluation as well as for label fusion (Figure 1). For details regarding the HD-GLIO processing pipeline, see Supplementary Appendix.

The output of HD-GLIO was compared to (1) the blinded manual delineations of each observer separately, emphasizing differences across disciplines, (2) the fused label, being the joint contribution from 2 disciplines, and (3) the aforementioned labels with added isotropic dilations of 5, 10, and 20 mm, representing margins clinically used in RT^9,10,26^ to assess any change in relative performance difference. Dice similarity coefficients were calculated as a geometric measure of spatial overlap between 2 segmentation masks. We also calculated Hausdorff Distance 95% (HD95) for calculation of the 95th percentile of the distances between boundary points in the 2 segmentation masks,^27^ details in Supplementary Appendix. We also assessed the volume size consistency between measures, as well as the sensitivity $\left(\frac{True\_Positive}{True\_Positive+False\_Negative}\right)$ and specificity $\left(\frac{True\_Negative}{True\_Negative+False\_Positive}\right)$ for each operator. As we had 2 operators to compare with HD-GLIO, we defined HD-GLIO as the reference segmentation and calculated metrics according to this definition.

### Prospective Validation on Clinical RT-Patients

HD-GLIO analyzed pre- and postoperative mpMRI-images (*n* = 20) acquired from 10 patients prior to radiation treatment planning. The mpMRI, with spatially coordinated HD-GLIO output volumes, were co-registered with the planning-CT in Eclipse treatment planning system. The HD-GLIO output volumes were applied as a prediction of RT GTV for clinical use, where preoperative HD-GLIO output volumes were selected for quantitative and qualitative evaluation for geometrical overlay according to ESTRO guidelines. Selection of preoperative HD-GLIO output volumes enabled comparable grounds for both CE- and NE-volumes. Postoperative HD-GLIO output volumes were selected for qualitative assessment according to RTOG guidelines that are based on delineation of the NE compartment, since contrast-enhancing tumor tissue was surgically removed. To prevent confirmation bias, the oncologist was blinded for the HD-GLIO-prediction until target delineation was performed. After target delineation, based on MRIs and planning-CT, the oncologist performed a clinical evaluation of the HD-GLIO-predictions. This was made according to a pre-defined qualitative scale ranging from 4 to 1 according to clinical utility,^28^ where: (4) “Contour requires no correction,” (3) “Contour requires only minor corrections, significant time saved,” (2) “Contour requires major corrections, little time saved,” and (1) “Contour not usable, no time saved.”

Dice scores between manual target volumes and preoperative HD-GLIO-prediction volumes were also calculated. HD-GLIO NE volumes were also qualitatively evaluated for geometric overlay on FLAIR- hyperintensity in postoperative mpMRIs.

All qualitative assessments were performed by a senior consultant oncologist with 14 years of experience.

### Clinical Response Evaluation

Responses to treatment were measured by clinical evaluation including (1) Neurological Assessment in Neuro-Oncology (NANO) scale, (2) scores on the EQ-5D-5L quality of life questionnaire, and (3) Karnofsky Performance Score, as described previously.^8^ Based on these assessments, the 13 patients were segregated into 3 groups (Table 1). Although survival data across groups were not statistically significant, difference in mean across groups supported clinical findings, as listed above. Group-1 was characterized by long survival and clinically stable disease, consisting of patients A, B, C, D, and E. Group-2 was characterized by short survival and rapid disease progression, consisting of patients F, G, H, I, and J. Group-3 was characterized by mixed clinical response and tumor doubling times, not fitting with characteristics in group-1 or group-2, and consisted of patients K, L, and M (details in Supplementary Table 1).

**Table 1.** BORTEM-17 Patient Characteristics

Characteristics		Retrospective			Prospective
		Group 1	Group 2	Group 3	Proof-of-concept^*^
Number of patients–*n*		*n* = 5	*n* = 5	*n* = 3	*n* = 10
Median age–years (Range)		54 (49–62)	52 (25–58)	34 (34–57)	66 (25–88)
Sex–*n* (%)	*Male*	3 (60.0)	4 (80.0)	2 (66.6)	7 (70.0)
	*Female*	2 (40.0)	1 (20.0)	1 (33.3)	3 (30.0)
IDH status–*n* (%)	*Mut*	0 (0.0)	2 (40)	1 (33.3)	3 (30.0)
	*Wt*	5 (100.0)	3 (60)	2 (66.6)	7 (70.0)
MGMT status–*n* (%)	*Methylated*	0 (0.0)	0 (0.0)	0 (0.0)	4 (40.0)
	*Unmethylated*	5 (100.0)	5 (100.0)	3 (100.0)	6 (60.0)
Median no. of BTZ cycle–*n* (range)		3 (2–6)	2 (2–4)	2 (2–2)	—
Median TMZ dose – mg/m^2^ (range)		175 (130–200)	200 (175–200)	187.5 (150–200)	—
Median Karnofsky performance status (range)		80 (80–100)	90 (80–100)	90 (80–90)	—
Median survival–months (range)		24.0 (13.4–41.0)	18.7 (11.2–32.4)	15.2 (12.8–39.9)	—
Median survival from recruitment–months (Range)		6.2 (2.5–20.9)	4.2 (2.4–15.9)	3.2 (2.2–23.8)	—

BORTEM-17 patient recruitment started in 2018 and were based on the 2016 WHO classification of glioblastoma.

^*^These patients were newly diagnosed with high-grade glioma, and served as controls for HD-GLIO clinical deployment in radiation therapy.

### Statistics

All statistics were analyzed with a 95% confidence interval using SPSS Version 26.0. (IBM, Armonk, NY). Dice similarity coefficients were computed with two-tailed Wilcoxon signed rank test. For volume size consistency assessment, the inter-item intra-class correlation coefficient (ICC) was calculated using two-way mixed model (details in supplementary appendix). For correlation between RANO measures and 3-dimensional CE volumes, we calculated Spearman’s non-parametric rank-order correlation coefficient with two-tailed test of significance, where 0 is no association and 1 is a perfect monotonic relationship, and >80 is regarded “very strong.”^29^ For correlation between CE- and NE-volumes, we used Pearson correlation with one-tailed test of significance. For difference in tumor volume regarding tumor location, we used linear mixed effect model, accounting for difference in number of repeated measures between patients.

## Results

Patient characteristics for all 23 patients are in Table 1. For detailed individual patient and tumor-compartment characteristics, please see Supplementary Tables 1 and 2, respectively.

### Dice- and HD95-Scores, Retrospective Dataset

HD-GLIO output volumes showed significant geometrical similarity with manual delineations.

**Figure 2.**
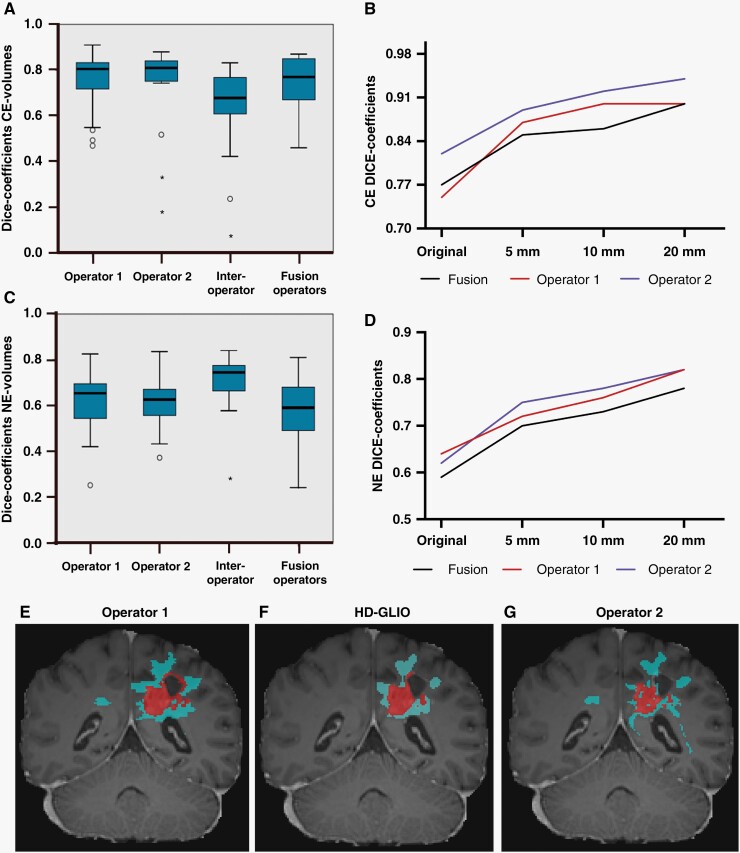
Volume similarity and discrepancy. (A, C) Boxplots of contrast-enhanced (CE)-volume and non-enhancing (NE)-volume Dice scores, respectively. (B, D) Line chart of median CE and NE Dice scores, respectively, after adding isotropic dilations. Red = operator-1, Blue = operator-2, and Black = fusion of operator-1- and operator-2 delineations. (C) Boxplots of NE-volume Dice scores. (E–G) Example of diverging delineations (Coronal view) where both operators (E and G) delineated a solitary island in the right hemisphere that HD-GLIO (F) excluded. Red segment = CE-volume, blue segment = NE-volume.

CE volumes showed median Dice scores of 0.81 (95% CI 0.71–0.83) and 0.82 (95% CI 0.74–0.84) for operator-1 and operator-2, respectively (Figure 2A). The median inter-operator Dice score for CE was 0.68 (95% CI 0.42–0.79). Fusion of operator-1 and operator-2 CE volumes showed median Dice scores of 0.77 (95% CI 0.64–0.85). The HD95 measurements for CE showed median of 5.91 (95% CI 2.8–16.4) and 3.16 (95% CI 2.8–7.1) for operator-1 and operator-2, respectively. The median inter-operator HD95 for CE was 8.5 (95% CI 2.8–32). Moreover, by dilating the 3D volumes by 5 mm or 10 mm, the relative performance difference across operators was reduced from 7% (baseline) to 2% (Figure 2B). When dilating the volume by 20 mm, in accordance with ESTRO guidelines,^9^ the median relative performance difference across operators was 4%. The median DICE scores improved significantly for both operators (*P* < .01), for all dilation sizes.

For NE volumes, median Dice scores were 0.65 (95% CI 0.56–0.69) and 0.63 (95% CI 0.57–0.67) for operator-1 and operator-2, respectively (Figure 2C). The median inter-operator score for NE was 0.74 (95% CI 0.58–0.78). Fusion of operator-1 and operator-2 NE volumes showed median Dice scores of 0.59 (95% CI 0.47–0.69). The HD95 for NE showed median of 16.1 (95% CI 10.6–22.2) and 16.7 (95% CI 9.4–23.2) for operator-1 and operator-2, respectively. The median inter-operator HD95 for NE was 6.39 (95% CI 2.8–13.0). Isotropic dilations of 20 mm equalized the relative NE-performance difference between operators (Figure 2D).

Taken together, we found that for CE, the Dice similarity and HD95 scored better between operator and HD-GLIO, than between operators. This indicates that HD GLIO predictions had a size, shape, and location that was intermediate between the operator-1 and operator-2 manual delineations. Adding dilations further increased Dice scores, and reduced the relative performance difference between individuals. For NE-volumes, Dice scores and HD95 showed poorer agreement between operator and HD-GLIO than between operator scores. This was because manual NE-delineations held substantially larger volumes than HD-GLIO predictions. For some patients, manual NE-delineations included isolated T2-FLAIR hyperintense regions, for example, in the contra-lateral hemisphere (Figure 2E–G), to a larger extent than computed by HD-GLIO. Both operators had specificity of 0.99 (95% CI = 0.99), respectively. Details regarding sensitivity in Supplementary Appendix.

### Prospective Validation on Clinical RT-Patients

**Figure 3.**
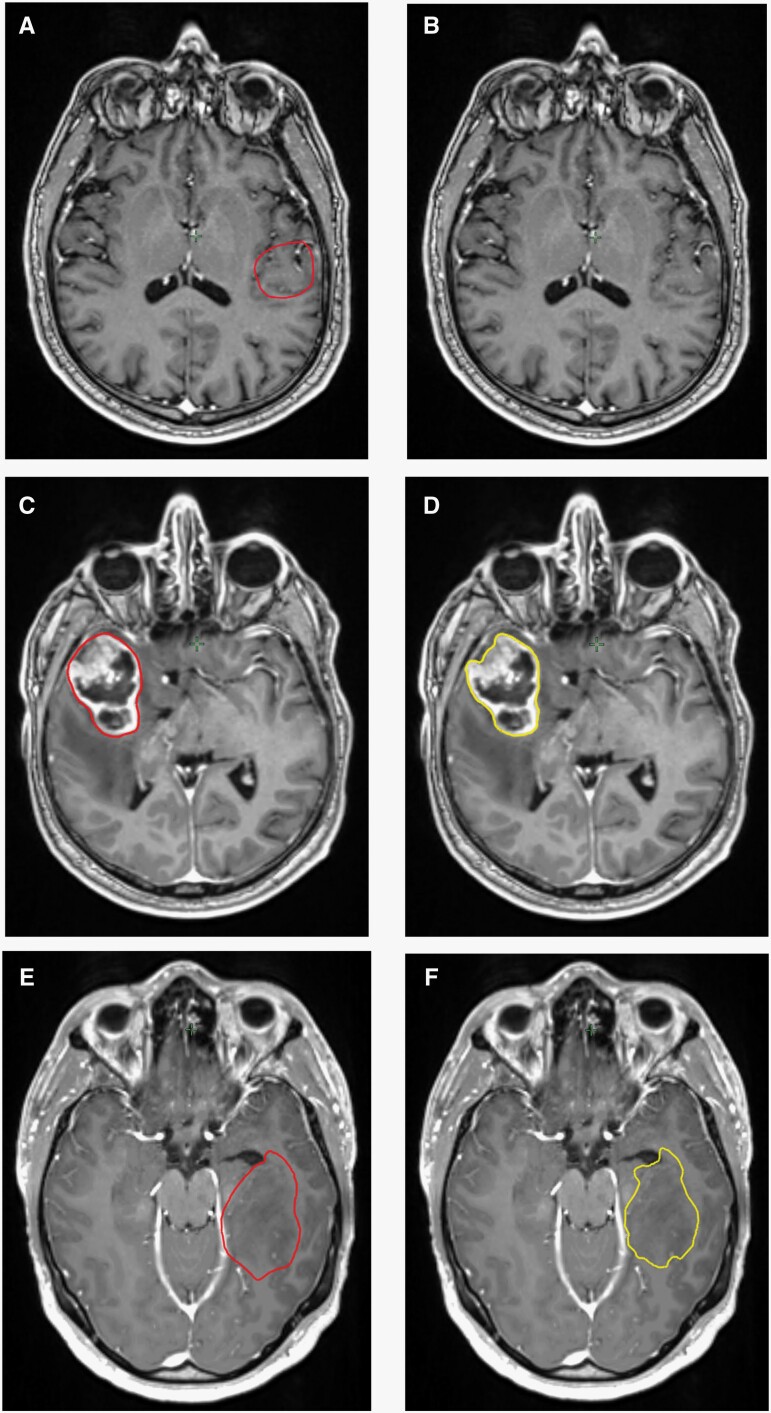
HD-GLIO predictions on clinical radiation therapy-patients. Red segment = manual gross target volume (GTV), Yellow segment = HD-GLIO prediction. (A) Manual GTV on patient holding atypical GBM expression, where (B) HD-GLIO failed to predict tumor volume. (C) Manual vs. (B) score-4 HD-GLIO prediction. (E) Manual GTV vs. (F) score-3 HD-GLIO prediction. All images: C-T1-w MRI.

**Figure 4.**
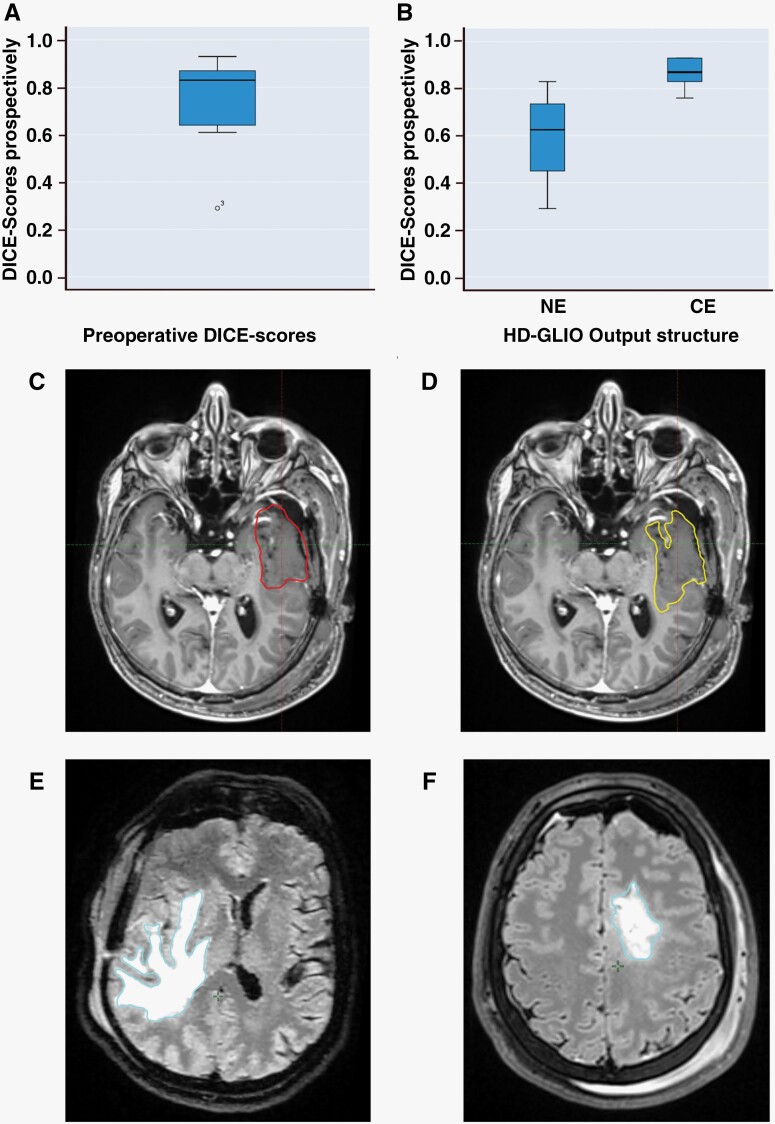
Prospective results. (A) Boxplot of overall DICE scores of preoperative prospective patients. (B) Boxplot of Dice scores according to prediction used. (C) Manual gross target volume (red segment) vs. (D) HD-GLIO (yellow segment) prediction demonstrating adaptions to nerves and vessels. (E-F) Postoperative non-enhancing predictions (blue segments) of FLAIR-hyperintensity volumes.

HD-GLIO successfully segmented pre- and postoperative grade-4 glioma tumor compartments in 9/10 cases. As expected, there was great heterogeneity in radiological expression, where preoperative mpMRIs from 5 patients revealed solid tumors with well-defined contrast enhancement and peritumoral edema. Four patients exhibited diffuse malignancy with limited contrast enhancement and some edema. One patient had an atypical radiological expression with no contrast enhancement and minimal edema, where HD-GLIO failed to predict any tumor (Figure 3A–B). Adaptations were made in which HD-GLIO output volume was found relevant for comparison corresponding to patient-specific radiological tumor characteristics, in concordance with ESTRO guidelines. CE volumes overlaid precisely with clinical volumes in tumors with solid contrast enhancement, while NE volumes were more accurate in diffuse tumor tissue. In the preoperative mpMRIs, HD-GLIO output could be used clinically without changes (score 4), on 2 patients (Figure 3C–D). HD-GLIO performed very well on 4 patients requiring only minor corrections (score 3) (Figure 3E–F), while on 2 patients, major corrections were required in parts of the target (score 2). Score 1—no time saved—was made on one patient. Overall, HD-GLIO showed a high degree of geometrical concordance with CTVs, with median Dice score of 0.83 (95% CI 0.59–0.90) (Figure 4A), in agreement with our retrospective analysis. Specifically, we observed that patients harboring tumors with well-defined contrast enhancement obtained median Dice-score of 0.87 (95% CI 0.77–0.95). This was significantly higher than diffuse tumors with median Dice-score of 0.63 (95% CI 0.24–0.95) (*P* < .05) (Figure 4B). This indicates that level of confidence is higher when a solid contrast-enhancing tumor is present. However, we observed that geometrical divergence between manual- and HD-GLIO volumes were partly due to HD-GLIO outlines being more complex in holding greater alignment with neighboring anatomical volumes, whereas manual delineations held smoother outlines, not avoiding adjacent vessels and nerves (Figure 4C–D). Taken together, preoperative HD-GLIO-outputs provided time-saving output volumes in 88% of cases, thus were deemed useful in a clinical setting.

All patients underwent gross total surgical resection. Postoperative mpMRIs showed minimal or no contrast enhancement on all patients, and large areas of hyperintense T2- FLAIR-signal on all patients except 1 patient exhibiting an atypical radiological expression. By qualitative assessment, all NE volumes were in concordance with true hyperintense T2- FLAIR-signal, requiring only minor corrections (score 3) (Figure 4E–F). Hence, postoperative NE volumes were useful for target delineation performed according to RTOG guidelines that include T2-FLAIR-hyperintensity in the GTV.

### MRI tumor burden assessment—volume size agreement

We assessed the agreement in volume (mL) among the 2 operators` delineations and the HD-GLIO segmentation. Inter-operator ICC was 0.65 for CE volumes, and 0.90 for NE volumes (*P* < .05, respectively). Furthermore, HD-GLIO yielded excellent CE-volume agreement with ICC = 0.90 and ICC = 0.95 for operator-1 and operator-2, respectively (*P* < .01, respectively). HD-GLIO also had excellent agreement for NE volumes, with ICCs of 0.97 and 0.90 for operator-1 and operator-2, respectively (*P* < .01, respectively).

For CE volumes, we also calculated ICC in relation to the RANO-prescribed measures, showing ICCs of 0.1, 0.15, and 0.2 for operator-1, HD-GLIO, and operator-2, respectively. RANO, based on volume estimation from 2-dimensional projections, was considerably smaller than the 3-dimensional measures, regardless of operator/HD-GLIO. Consequently, ICCs showed overall poor agreement with the RANO measures. Therefore, we assessed the Spearman correlation between RANO measures and the 3-dimensional CE volumes. There was a strong positive correlation between RANO and HD-GLIO (Spearman R^2^ = 0.83 *P* < .001), and with RANO and operator-2, R^2^ = 0.80 (*P* < .001). Correlation with operator-1 was not statistically significant.

In summary, HD-GLIO tumor burden assessment concurred with both operators, and HD-GLIO volume sizes held a clear proportional relationship with RANO volumes.

### Longitudinal Features in MRI-Measurements in Relation to Clinical Response

**Figure 5.**
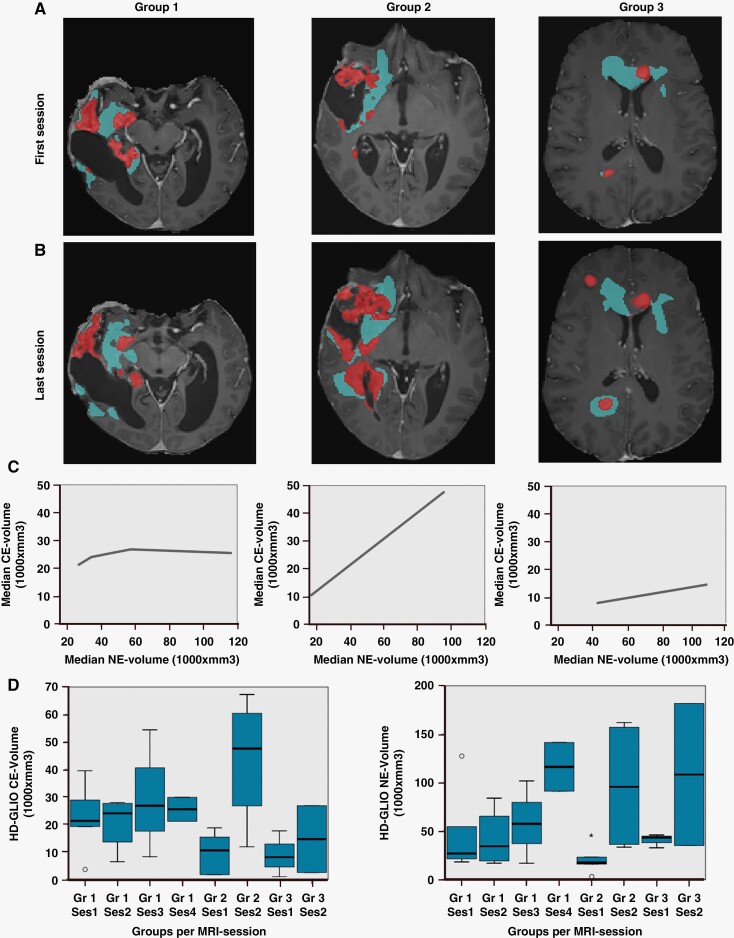
Tumor compartment growth patterns between groups. (A-B) contrast-enhanced (CE)-volumes (red segments) and non-enhancing (NE)-volumes (blue segments) longitudinal development for patients across groups. (C) Group-wise longitudinal development in median CE-volume (y-axis) along NE-volume (x-axis). (D) Boxplots of group-wise longitudinal development of CE-volumes (left) and NE-volumes (right).

The patients were partitioned a priori into 3 groups according to clinical response criteria.^8^ We heuristically screened CE- and NE-volumes in search of discriminative features in the longitudinal mpMRI recordings that could be related to clinical response in our cohort (Figure 5A–B). As both operators had good or excellent ICC with HD-GLIO for both CE- and NE-volumes, we found HD-GLIO volume sizes (*n* = 58, ie, 29 exams × 2 compartments) and volume changes to be candidate for further investigation regarding reported clinical response.

For group-1 (number of repeated measures = 14) we had 4 MRI sessions with 56 days interval. These patients demonstrated responses to treatment and the longest overall survival, Supplementary Table 1. When comparing the first 2 sessions, we found that the NE compartment volume increase was least prominent in group-1 among the 3 groups (Figure 5C, D). Moreover, in this group, median CE volumes were reduced from third to last time point. Patients in group 2 had such rapid progression they did not live to their third MRI session. Group-2 (number of repeated measures = 9) had the smallest initial NE volumes, but showed the steepest increase in both median CE-volume and median NE-volume across all 3 groups, (Figure 5 C). For group-3 (number of repeated measures = 5), increase in median CE and median NE volumes was intermediate. Hence, the ratio (median NE-volume/ median CE-volume) differed between the groups. We calculated Pearson correlation as a measure of the strength of the linear relationship between the CE- and NE-volumes for each group and found Pearson correlations of 0.55 (*P* = .02), 0.91 (*P* < .01), and 0.80 (*P* = .05) for group-1, 2, and 3, respectively.

Disregarding the groups, we screened for other factors related to tumor volume. We performed a linear mixed model analysis and observed that CE/NE-relations corresponded with tumor location, where right-hemisphere tumors obtained larger volumes (mean 27 361 mL) than left-hemisphere (mean 15 154 mL) (*P* = .02), Supplementary Table 2. Pearson correlations were 0.72 (*P* < .01) and 0.86 (*P* < .01) for right- and left-sided tumors, respectively.

## Discussion

The objective of this study was to investigate the feasibility of clinical deployment of HD-GLIO as a support tool in radiotherapy target delineation, for radiological tumor burden assessment, and to screen for discriminative features in longitudinal MRI data that may be correlated to clinical treatment response. The major findings were: (1) HD-GLIO showed significant geometrical similarity with manual delineations and saved time during tumor volume prediction for clinical radiation treatment planning, (2) HD-GLIO had strong statistical correlation with RANO, and (3) longitudinal CE/NE-volume growth rate differed significantly in relation to treatment response. Prospective findings were that CE volumes held high geometrical overlap with clinical volumes in tumors with solid contrast enhancement, while NE volumes held more accurate overlap in diffuse tumor tissue.

As expected and confirming the motivation for this study, we found substantial variability between clinical operators for both Dice scores and volume sizes. This could be due to extrinsic factors such as the operators’ experience and subjective perception characteristics, or, intrinsic factors contributing to radiological interpretation complexity, such as immune response, surgery, effects of chemotherapy, and previous radiotherapy. However, in the training of computer-based DL models, expert knowledge from years of radiological interpretation training can be transformed into automated pattern recognition tools. Such knowledge transfer can promote standardization of tumor volume annotation, thereby reducing variation in patient care. Moreover, the clinical tolerance of inter-operator variability can help set the standard for acceptable variability between AI predictions and expert delineations.

### Limitations of the Study

When comparing new methods to the ground truth, uncertainty remains regarding false positive/false negative tumor voxels. This constrains validation of AI models in cases where AI challenges or outperforms the ground truth. Furthermore, limitations in segmentation performance will depend on the external validity of the training data, where potential underrepresentation of certain tumor expressions in training data could lead to false positive or false negative detection of tumor burden. Although part of our study entailed retrospective analyses with inherent confounders such as surveillance bias and varying imaging time points, validation with a small but independent prospective cohort did confirm the findings. The small sample size of the prospective cohort may have limited external validity, in particular when radiological expression within the group is highly heterogeneous. In these prospective patients, preoperative tumor outlines largely had good intracranial spatial concordance with postoperative tumor cavities. Thus, preoperative tumor geometry remained spatially relevant when fused with planning CT. Further validation in a larger, randomized cohort, including several hospital systems and geographical regions with more diverse populations, or by incorporating larger datasets is required.

### Feasibility for HD-GLIO in Radiotherapy Target Delineation

Recent development in photon and proton RT techniques with improved target coverage and steep dose gradients enable better organ-at-risk dose sparing and have further advantage of high-precision target delineation. We addressed the use of mpMRI and the pre-trained HD-GLIO ensemble model to determine if this can aid in target volume delineation on the patients undergoing treatment in the phase 1B/II BORTEM-17 clinical trial (ClinicalTrials.gov Identifier: NCT03643549).

Retrospective median Dice scores for the CE volumes, 0.81 and 0.82 for operator-1 and operator-2, respectively, were considerably better than median inter-operator Dice score of 0.68, in concordance with HD95 measures. Prospective validation of oncologist agreement with HD-GLIO confirmed retrospective findings, showing median Dice score of 0.87. Moreover, the oncologist’s clinical evaluation was that the HD-GLIO-predicted CE volume was clinically usable in all cases, contributing to a more efficient workflow.

Retrospective Dice scores for NE in this study were somewhat poorer than for CE, and when manually inspecting the NE-volume discrepancies, we observed HD-GLIO tends to segment slightly (≈1 mm) more restricted than manual delineations along the outline. This could be due to visual perception characteristics as well as physical precision in manual delineations. Moreover, for some patients, manual delineations included solitary islands of hyperintense T2-FLAIR-signal that HD-GLIO excluded. There are, however, no definite data suggesting such inclusion alters patient outcome, which is why ESTRO-ACROP guidelines advise not to include all hyperintense T2-FLAIR regions on primary grade IV tumors.^9,11^ Prospective validation of preoperative NE-volumes confirmed retrospective Dice-score of 0.63. However, qualitative scores showed that the NE volumes still were found useful for majority of cases, in terms of potential time saved. The oncologist also reported that HD-GLIO-predictions could represent quality assurance for clinicians in training or activate reassessment in complex cases. The qualitative and quantitative scores corresponded.

Prospective results demonstrate that even though some variations were seen, overall HD-GLIO performance was found useful for clinical deployment. Best results were found for CE volumes, where clinical utility was also most readily recognized due to the well-defined contrast enhancement. Our NE results showed more variation, and held lower scores than CE. This is not unexpected, as tumors exhibiting diffuse radiological expressions can be more challenging to define. Thus, a more laborious assessment is required for this group, which might be reflected by the potential time saved. NE volumes were most useful when evaluated postoperatively in relation to RTOG guidelines. This could be related to T2-FLAIR-hyperintensity region being more straightforward to assess, compared to ESTRO guidelines depending on more complex assessment across the mpMRI.

Nevertheless, the HD-GLIO tool demonstrated ability to segment vector-valued voxels in mpMRI expressing high-dimensional tumor tissue signatures with geometrical similarity for both CE and NE. This was with the use of standard brain mpMRI acquisition protocol. We have also shown that the relative performance difference between HD-GLIO predictions and manual delineations is significantly reduced when isotropic margins are added, increasing the relevance of RT target delineation. However, we emphasize that the final design of the RT target volumes will depend on the treating oncologist, and that radiological data should be interpreted according to salient clinical information.

### MRI tumor burden assessment—volume size agreement

Limited time resources have been the point of contention for not implementing 3-dimensional tumor volume assessment in clinical routine, even though the consensus is that 3D gives the most accurate measure.^19,30^ For a single examination, the complete HD-GLIO pipeline (including spatial alignment of the channel images, brain extraction, and segmentation) took less than 4 minutes wall time, providing CE+NE tumor compartments with instant volumetric calculation in good agreement with expert delineations. When we assessed the volume size correlation between HD-GLIO and operators, main ­findings showed that HD-GLIO had overall excellent reliability with both operators. For both CE and NE, reliability in size between HD-GLIO and operators were superior to the inter-operator reliability. We also found a very strong correlation between HD-GLIO and the current gold standard RANO measurements. However, RANO volumes were significantly smaller than 3-dimensional volumes, implying the 2-dimensional RANO measures severely underestimate the anisotropic tumor volume.

### Longitudinal Features Correlated to Clinical Response

We previously reported^8^ that our cohort was segregated into 3 groups of diverging clinical response measures; responders (group-1), non-responders (group-2), and mixed/intermediate response (group-3). In this study, we investigated whether there may be discriminative features in the longitudinal MRI data that correlated to these clinical treatment responses by assessing the relationship of CE- and NE-volumes in the 3 groups, as well as tumor location. We found that group-1 patients, who exhibited good clinical response to treatment, had the smallest linear relationship between CE- and NE-volumes (Pearson correlation = 0.55). This means that angiogenetic tumor growth was restricted, and that the biological processes inducing edema and hyperintense T2-FLAIR-signal, developed more slowly than for the other groups. This was also the group characterized by a more activated immune response.^8^ Group-2 patients exhibited a very strong linear relationship between CE- and NE-volume increases (Pearson correlation = 0.91), and was the group with poorest response to treatment, as well as lowest OS. The difference in relational tumor-compartment volume development fits well with difference in clinical response for the patients in our study. This relational growth also correlated with tumor location, where right-hemisphere tumors had larger volume than left-hemisphere tumors, in concordance with findings in other studies.^31^

Taken together, this could indicate that increase in NE-volume relative to CE- volume i.e, tumor-compartment growth correlation, is a prognostic factor (proxy indicator) for treatment response. To the best of our knowledge, there have not been other studies assessing this relationship, although peritumoral edema has been suggested as a negative prognostic factor for survival in GBM.^32^ The incorporation of T2-FLAIR-changes in the determination of progression was one of the major changes proposed by the RANO criteria for high-grade gliomas; however, because of the difficulty of measuring T2-FLAIR hyperintensity volume accurately, no objective criteria were proposed.^15^ In this study, we found the HD-GLIO model to be an effective tool in quantifying 3D tumor volume on repetitive measures, where the dual-compartment assessment adds value to the clinical usefulness. Adding weight to radiological tumor burden as a marker of response also increases objectiveness by reducing impact of subjective interpretation-diversity bias in Patient Reported Outcome Measures.

Although our data regarding characteristic group-wise growth pattern features are somewhat limited by the small sample sizes across groups, we will prospectively investigate this CE/NE tumor-compartment growth correlation further as more patients are included in the ongoing study.

## Conclusions

In our study, comprising 23 patients and 49 MRI examinations, HD-GLIO DL predictions demonstrated high agreement (Dice similarity score and HD95) with manual delineations in segmenting grade 4 glioma tumor compartments on multi-parametric MRI. Our data indicate that the HD-GLIO tool could serve as a first step in target volume delineation for radiotherapy, where the computed CE- and NE-compartments would be subsequently assessed by the oncologist, being responsible for the final design of GTV and CTV.

We found a strong correlation to volumetric RANO measures, suggesting HD-GLIO is feasible for MRI-based 3D tumor burden assessment in clinical decision-making as well as in trials.

We also suggest the tumor-compartment growth correlation (longitudinal CE/NE volume ratio) could serve as a prognostic factor for treatment response. An ongoing prospective study is investigating this tumor-compartment growth correlation to validate its prediction for treatment response in larger sample and independent population.

## Supplementary Material

vdad037_suppl_Supplementary_Appendix

vdad037_suppl_Supplementary_Data

## Data Availability

Data cannot be shared publicly because this is sensitive patient data. Data are available from the corresponding authors (MC) as they are approved by the Ethics Committee (approval number 2017/2084/REK) for researchers who meet the criteria for access to confidential data.
