# Supplementary material for: Feasibility of deep learning-based tumor segmentation for target delineation and response assessment in grade-4 glioma using multi-parametric MRI
Source: Neurooncol Adv. 2023 Apr 13;5(1):vdad037. doi: 10.1093/noajnl/vdad037 (PMC10162115; doi:10.1093/noajnl/vdad037)
Supplement: vdad037_suppl_Supplementary_Appendix [file vdad037_suppl_supplementary_appendix.docx]

Feasibility of deep learning-based tumor segmentation for target delineation and response assessment in grade 4 glioma using multi-parametric MRI

Authors: Marianne H Hannisdal, Dorota Goplen, Saruar Alam, Judit Haasz, Leif Oltedal, Mohummad A Rahman, Cecilie Brekke Rygh, Stein Atle Lie, Arvid Lundervold, and Martha Chekenya

**Supplementary appendix**

*HD-GLIO pipeline*

Pre-processing of the images included (i) re-slicing the multiple MRI sequences to the one with highest spatial resolution (T1-w); (ii) reorient the resliced sequences and the T1-w series to standard (MNI152) space; (iii) scull stripping of each reoriented image using HD-BET (https://github.com/MIC-DKFZ/HD-BET); (iv) register all scull-stripped images to the template (T1-w) sequence using 6 DOF affine transformation with spline interpolation; (v) reapply the T1-w brain mask to force non-brain voxels to be 0. Finally, we applied HD-GLIO on the scull-stripped registered, multi-parametric image (T1-w, cT1-w, T2-FLAIR, T2-w). The complete and fully automated HD-GLIO pipeline for segmenting the CE and NE components of the tumor with calculation of their volumes [microL] and color-coded visualizations was implemented in a Jupyter notebook accessing all necessary tools (FreeSurfer, FSL) and Python libraries (e.g. numpy, matplotlib, nibabel, nilearn, MONAI/PyTorch) being installed in a separate conda environment. Using a Dell Precision laptop running Ubuntu 18.04, with an 8-core Xeon processor, 64 GB memory and an NVIDIA RTX 3000 6 GB GPU, the processing time was approximately 4 min per examination.

*Manual delineation*

Operator-1 delineated CE and NE on 22 of the 29 datasets, Operator-2 delineated CE and NE on 16 of the 29 datasets, whereof 14 datasets were overlapping between the two operators. The operators were blinded for the HD-GLIO output as well as the output from the other operator.

*Hausdorff Distance 95*

Hausdorff distance 95% (HD95), being a robust version of the Hausdorff distance (HD) i.e. the maximum distance of a segmented mask to the nearest point in the other segmented mask, where HD95 is based on the calculation of the 95^th^ percentile of the distances between boundary points in the two segmentation masks, a metric of local segmentation agreement that eliminates the impact of a very small subset of the outliers (local disagreements) ^1^.

*ICC*

ICC (consistency type) were computed by individual calculations of the inter-operator ICC, and ICCs between each operator and HD-GLIO, respectively. In this context ICC is a metric of consistency between operators and machine measuring the same quantity, i.e. performing the same segmentation task regarding CE and NE in a given dataset, where the ICC estimator can be defined in terms of a linear mixed effects model. Heuristic guidelines ^2^ for the interpretation of ICC inter-operator agreement measures are: ICC < 0.5 [poor], 0.50 <= ICC < 0.75 [moderate], 0.74 <= ICC < 0.90 [good], ICC >= 0.90 [excellent].

*Specificity and sensitivity*

The Dice scores represent the sum of specificity and sensitivity. On our retrospective dataset, we found that Operator-1 had a sensitivity of 0.72 (95% CI 0.59-0.77) and 0.53 (95% CI 0.43-0.57) for the CE and NE compartments, respectively. Operator-2 had a sensitivity of 0.89 (95% CI 0.87-0.92) and 0.54 (95% CI 0.48-0.63) for CE and NE volumes, respectively. Both operators had specificity of 0.99 (95% CI=0.99), respectively (Supplementary Figure 2).

1. Mattila P. Hausdorff dimension of projections and distance sets. *Fourier Analysis and Hausdorff Dimension*. Cambridge University Press; 2015:55-71.

2. Koo TK, Li MY. A Guideline of Selecting and Reporting Intraclass Correlation Coefficients for Reliability Research. Lombard, IL :2016. p. 155-163.

**Supplementary Figure 1**


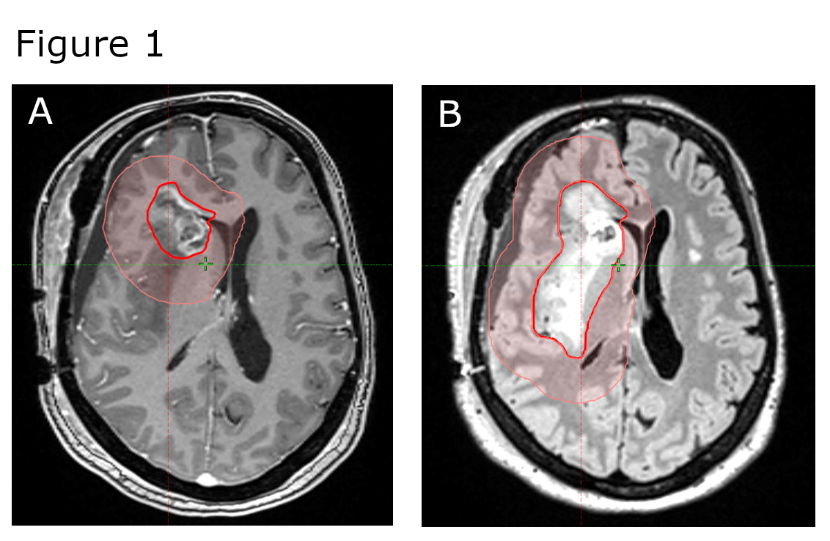


Supplementary Figure 1: Illustration of the volumetric implication of target delineation following the two guidelines: A: ESTRO-ACROP guidelines: contrast-enhanced T1-image illustrating ESTRO-ACROP guidelines delineation of GTV (red middle contour) including only T1 contrast-enhancing tumour, with 2cm surrounding CTV-margin (pink pseudo-color coded outer segment). B: RTOG guidelines; T2FLAIR-image illustrating RTOG guidelines for delineation of GTV (red middle contour), including both T1 contrast-enhancing tumour and T2FLAIR abnormality/oedema, with 2cm surrounding CTV-margin (pink pseudo-color coded outer segment). (Illustration: in-study patient, Eclipse Aria Oncology Information Systems (Varian, California, USA)).

**Supplementary Figure 2**


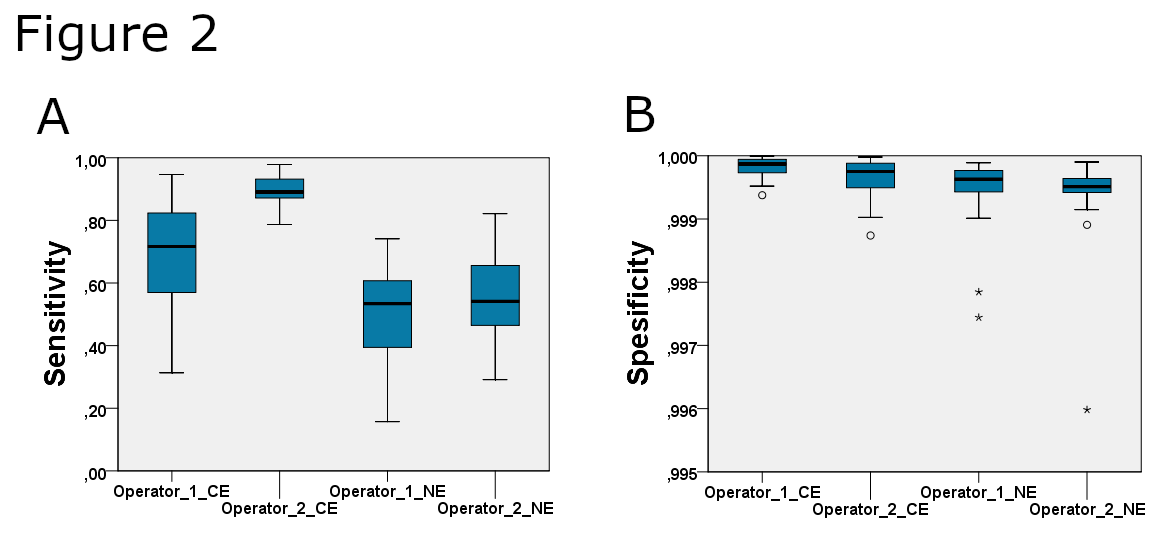


Supplementary Figure 2: Boxplots of (A) Sensitivity scores of Operator1 and Operator 2 for CE and NE, respectively, denoting the cocurrance with HD-GLIO in identifying voxels with disease, and (B) Spesificity scores of Operator1 and Operator 2 for CE and NE, respectively, denoting the cocurrance with HD-GLIO in identifying voxels without disease. Box show IQR of datapoints, with the horizontal central line showing the median. Whiskers adjacent to the boxes represent 1·5 times the IQR. Dots are outliers, ° representing mild outliers, * representing extreme outliers. All results from retrospective dataset.

**Supplementary Table 1**: BORTEM-17 individual patient characteristics

|  | **Patient ID** | **Age** | **Sex** | **KPS** | **IDH**  **Status** | **# of  BTZ cycle** | **Median TMZ  dose mg/m2** | **# of MR** | **Overall**  **Survival  (months)** | **Survival after  Recruitment (months)** | **L/D Status** | **Treatment  Response** |
| --- | --- | --- | --- | --- | --- | --- | --- | --- | --- | --- | --- | --- |
| Group 1  Long Survival,  objective response | A | 53 | Male | 80 | Wt | 2 | 162.5 | 1 | 24.0 | 6.2 | Dead | - |
|  | B | 53 | Male | 80 | Wt | 6 | 200 | 3 | 33.8 | 14.5 | Dead | PR |
|  | C | 62 | Female | 100 | Wt | 3 | 175 | 2 | 13.4 | 5.4 | Dead | PR |
|  | D | 49 | Male | 80 | Wt | 5 | 150 | 4 | 41.0 | 20.9 | Dead | SD |
|  | E | 55 | Female | 80 | Wt | 2 | 140 | 3 | 23.7 | 2.5 | Dead | SD |
| Group 2  Short survival, rapid progression | F | 39 | Female | 90 | Mut | 2 | 187.5 | 2 | 13.8 | 3.8 | Dead | - |
|  | G | 25 | Male | 100 | Mut | 2 | 187.5 | 2 | 32.4 | 15.0 | Dead | - |
|  | H | 56 | Male | 80 | Wt | 2 | 200 | 1 | 18.7 | 4.2 | Dead | - |
|  | I | 52 | Male | 80 | Wt | 2 | 200 | 2 | 11.2 | 2.4 | Dead | - |
|  | J | 58 | Male | 90 | Wt | 4 | 200 | 2 | 25.9 | 8.2 | Dead | - |
| Group 3  Mixed survival and progression | K | 34 | Male | 90 | Mut | 2 | 187.5 | 3* | 39.9 | 23.8 | Dead | - |
|  | L | 34 | Female | 80 | Wt | 2 | 150 | 2 | 15.2 | 3.2 | Dead | - |
|  | M | 57 | Male | 90 | Wt | 2 | 200 | 1 | 12.8 | 2.2 | Dead | - |
| Prospective cohort for “proof-of-concept” | N | 88 | Male | - | Wt | - | - | - | - | - | - | - |
|  | O | 50 | Female | - | Mut | - | - | - | - | - | - | - |
|  | P | 71 | Male | - | Wt | - | - | - | - | - | - | - |
|  | Q | 66 | Female | - | Wt | - | - | - | - | - | - | - |
|  | R | 62 | Male | - | Wt | - | - | - | - | - | - | - |
|  | S | 25 | Male | - | Mut | - | - | - | - | - | - | - |
|  | T | 71 | Male | - | Wt | - | - | - | - | - | - | - |
|  | U | 38 | Male | - | Mut | - | - | - | - | - | - | - |
|  | V | 66 | Male | - | Wt | - | - | - | - | - | - | - |
|  | W | 68 | Female | - | Wt | - | - | - | - | - | - | - |

KPS- Karnofsky performance status, IDH- isocitrate dehydrogenase, Mut- Mutant, Wt- Wild type, BTZ- Bortezomib, TMZ- Temozolomide, L/D- Live/Dead, PR- Partial response, SD- Stable disease.

**Supplementary Table 2**: Tumor compartment characteristics

| **Case** | **Primary Tumor Site** | **CE-volume microL** | **CE-voxel intensity Mean ± SD *** | **NE-volume microL** | **NE-voxel intensity Mean ± SD **** | **KPS** |
| --- | --- | --- | --- | --- | --- | --- |
| A_session01 | Right Frontal | 19215.00 | 606.8028±147.1529 | 21345.00 | 202.7509±16.9223 | 80 |
| A_session02 |  | 20722.00 | 486.1327±104.8925 | 16797.00 | 182.5654±17.8778 | 60 |
| B_session01 | Left Parietal | 3560.00 | 468.0668±75.7647 | 18163.00 | 186.2675±17.0644 | 80 |
| B_session02 |  | 6403.00 | 443.6547±70.4585 | 21824.00 | 190.5212±20.4677 | 80 |
| B_session03 |  | 8204.00 | 419.1304±65.1954 | 16734.00 | 183.0823±18.5102 | 80 |
| B_session04 |  | 21102.00 | 450.1875±91.4839 | 91178.00 | 191.1779±18.2262 | 80 |
| C_session01 | Left Temporal | 21251.00 | 523.3960±138.6332 | 26902.00 | 162.8068±12.4196 | 100 |
| D_session01 | Left Temporal | 39506.00 | 515.8213±111.6232 | 127457.00 | 191.7481±22.5402 | 80 |
| D_session02 |  | 27303.00 | 576.1002±135.9852 | 83976.00 | 210.4797±22.2696 | 80 |
| D_session03 |  | 26784.00 | 513.0891±132.7145 | 101779.00 | 185.9869±22.6108 | NA |
| D_session04 |  | 29820.00 | 538.1744±134.7310 | 141500.00 | 174.8379±19.2401 | 80 |
| E_session01 | Right | 28841.00 | 571.0097±103.6172 | 54538.00 | 180.7282±9.7692 | 80 |
| E_session02 |  | 27968.00 | 503.8331±96.2031 | 46857.00 | 176.5576±10.3937 | 60 |
| E_session03 |  | 54411.00 | 533.8062±104.5969 | 57481.00 | 169.1040±11.4832 | NA |
| F_session01 | Right Temporal | 10525.00 | 592.3621±117.4020 | 23041.00 | 217.0247±17.0035 | 90 |
| F_session02 |  | 41658.00 | 615.6301±114.5264 | 39133.00 | 208.5387±16.3139 | 80 |
| G_session01 | Right Frontal | 18699.00 | 425.8892±93.6803 | 45750.00 | 180.0962±13.5012 | 100 |
| G_session02 |  | 53534.00 | 520.7463±119.4937 | 152276.00 | 193.0592±14.6205 | 90 |
| H_session01 | Left Frontal | 1676.00 | 415.6246±48.6918 | 17474.00 | 201.6851±14.6613 | 80 |
| I_session01 | Right Parietal | 15369.00 | 512.8739±119.9472 | 15883.00 | 205.2946±27.5119 | 80 |
| I_session02 |  | 78410.00 | 743.0404±213.0434 | 188887.00 | 234.7877±21.0353 | NA |
| J_session01 | Right Frontal | 1601.00 | 468.5255±88.9923 | 2934.00 | 167.9220±14.6910 | 90 |
| J_session02 |  | 11809.00 | 589.5779±124.4662 | 33421.00 | 192.7869±15.6524 | 90 |
| K_session01 | Right Frontal | 17614.00 | 482.2399±115.7774 | 43386.00 | 182.2229±14.4678 | 90 |
| K_session02 |  | 26771.00 | 529.9569±143.7651 | 181720.00 | 209.0877±19.5477 | 80 |
| K_session02 |  | 10638.00 | 538.5555±150.1489 | 25651.00 | 186.3338±16.9806 | 90 |
| L_session01 | Left Parietal | 923.00 | 555.1933±129.0834 | 46059.00 | 194.7338±15.1688 | 80 |
| L_session02 |  | 2444.00 | 548.8468±102.3918 | 35117.00 | 189.4004±11.2676 | 60 |
| M_session01 | Left Temporal | 8032.00 | 600.3048±135.5820 | 32730.00 | 201.3630±17.3301 | 90 |

* CE-voxel intensity on contrast-enhanced T1-weighted image, ** NE-voxel intensity on Fluid Attenuated Inversion Recovery image. NA=not available. Volume and voxel intensities of HD-GLIO output compartments. All patients harbored unmethylated MGMT-status.
