# Supplementary material for: Feasibility of deep learning-based tumor segmentation for target delineation and response assessment in grade-4 glioma using multi-parametric MRI
Source: Neurooncol Adv. 2023 Apr 13;5(1):vdad037. doi: 10.1093/noajnl/vdad037 (PMC10162115; doi:10.1093/noajnl/vdad037)
Supplement: vdad037_suppl_Supplementary_Data [file vdad037_suppl_supplementary_data.pdf]

HD-GLIO-BORTEM-PSEUDONYM

A.L. @ MMIV-ML

Installation regarding RTX 3090 (and A5000):

```
Note: GeForce RTX 3090 with CUDA capability sm_86 is not compatible with the
current PyTorch installation.
      The current PyTorch install supports CUDA capabilities sm_37 sm_50 sm_
60 sm_70
      If you want to use the GeForce RTX 3090 GPU with PyTorch,
      please check the instructions at https://pytorch.org/get-started/local
ly
```

Remove previous `bortem` environment

```
conda deactivate
conda env remove -n bortem
```

Reinstall `bortem` environment with new Pytorch and Cuda toolkit 11.1 to run on RTX 3090

```
conda create -n bortem python=3.8 ipython -y && conda activate bortem
conda install jupyter
conda install pytorch torchvision torchaudio cudatoolkit=11.3 -c pytorch
conda install pytorch-geometric -c rusty1s -c conda-forge
python -m ipykernel install --user --name bortem --display-name "BORTEM"
conda install scikit-image
```

Then the rest of the `bortem` environment according to `environment.yml`

```
conda env update
```

Check use of GPUs (TITAN V and RTX 3090) with

```
watch -n 0.1 nvidia-smi
```

HD-GLIO information

Philipp Kickingereder, Fabian Isensee, et al. Automated quantitative tumour response assessment of MRI in neuro-oncology with artificial neural networks: a multicentre, retrospective study. Lancet Oncol 2019; 20: 728–740.
https://www.thelancet.com/journals/lanonc/article/PIIS1470-2045(19)30098-1/fulltext
(https://www.thelancet.com/journals/lanonc/article/PIIS1470-2045(19)30098-1/fulltext)

Segmentation info:

- **CEs**: contrast-enhancing tumours (mask=1)
- **NEs** (non-enhancing T2-signal abnormalities, mask=2): defined as T2-FLAIR hyperintense abnormality excluding the contrast-enhancing and necrotic portion of the tumour, resection cavity, and obvious leukoaraiosis.

- Delineated volumetrically by experienced neuroradiologists
- We defined **tumour progression** as an increase in tumour volume (compared with baseline or best response) in either CE tumour or NEs, beyond a minimal tumour volume of 1 cm^3; or occurrence of a new CE lesion outside of the CE tumour volume from the previous MRI scan (identified automatically using dedicated algorithms with the respective segmentation masks over time as input (cf. the appendix)

In [ ]:

```
%matplotlib inline
# This to be able to display figures and graphs within the notebook browser

import os
import os.path as op
import shutil
import subprocess as subp
import pathlib
import glob
import shutil
import string
from datetime import date
import warnings
import numpy as np
import pandas as pd
import nibabel as nib
from nibabel.viewers import OrthoSlicer3D
import scipy
from nilearn import image
from nilearn import plotting
from nilearn.plotting import plot_roi
import matplotlib.pyplot as plt
from matplotlib.colors import LinearSegmentedColormap
import seaborn as sns
from nilearn.image.image import mean_img
from sklearn.cluster import KMeans
from nilearn.masking import apply_mask
from sklearn.preprocessing import StandardScaler
from sklearn.neighbors import KNeighborsClassifier
from sklearn.metrics import classification_report, confusion_matrix
from sklearn.ensemble import RandomForestClassifier
import IPython
from matplotlib.colors import ListedColormap
```

In [ ]:

```
home = os.path.expanduser('~')


TREE = '/usr/bin/tree'

MRICONVERT = '/usr/local/freesurfer/7.3.2/bin/mri_convert'
FSLREORIENT2STD = '/usr/local/fsl/bin/fslreorient2std'
FLIRT = '/usr/local/fsl/bin/flirt'
FSLMATHS = '/usr/local/fsl/bin/fslmaths'

HDBET = f"{home}/anaconda3/envs/bortem/bin/hd-bet"
HDGLIO_PRED = f"{home}/anaconda3/envs/bortem/bin/hd_glio_predict"


base_dir = f"{os.path.expanduser('~')}/prj/Bortem"
nifti_dir = f"{base_dir}/Bortem_nifti_for_hdglio_pseudonym"
synthseg_dir = f"{base_dir}/Bortem_synthseg_pseudonym"
test_hd_glio = f"{base_dir}/Bortem_test_hdglio_pseudonym"
results = f"{base_dir}/Bortem_hdglio_pseudonym"


all_chns = ['T1', 'CT1', 'T2', 'FLAIR']
all_chns_synthseg = ['T1', 'CT1', 'T2', 'FLAIR', 'T1_synthseg_parc_robust']
template = 'T1'
inp_chns = [c for c in all_chns if c != template]

# AD HOC colormap for HD-GLIO  (CE, NE)-segmentation overlay
# colors = [(1, 0, 0),  (0, 1, 0), (0, 0, 1)]  # R -> G -> B
colors = [(1, 0, 0), (0, 0, 1), (0, 1, 0)]  # R -> B -> G
n_bins = [2, 3, 6, 10, 100]  # Discretizes the interpolation into bins
cmap_name = 'my_cm'
cm = LinearSegmentedColormap.from_list(cmap_name, colors, N=n_bins[0])
```

In [ ]:

```
os.environ['MKL_THREADING_LAYER'] = 'GNU'
import torch
torch.cuda.is_available()
```

In [ ]:

```
!nvidia-smi
```

Get a list of all exams in `nifti_dir`

In [ ]:

```
cmd = [TREE, nifti_dir]
cmd_str = " ".join(cmd)
```

In [ ]:

```
%%bash -s "$cmd_str"
$1
```

In [ ]:

```
cmd = ['du', nifti_dir]
cmd_str = " ".join(cmd)
```

In [ ]:

```
%%bash -s "$cmd_str"
$1 -hk --max-depth=2 | sort -k2
```

Make helper functions

In [ ]:

```
import warnings
warnings.filterwarnings("ignore")
```

AD HOC: Get image matrices and voxel resolutions for each channel

In [ ]:

```
def get_shape_res(base, exam):  # sub, ses):

    sub = exam[0:1]
    ses = exam[2:4]
    fnbase = f"{base}/{exam}/{sub}_{ses}"
    T1fn = f"{fnbase}_T1.nii.gz"          # T1.nii.gz
    CT1fn = f"{fnbase}_CT1.nii.gz"         # CT1.nii.gz
    T2fn = f"{fnbase}_T2.nii.gz"           # T2.nii.gz
    FLAIRfn = f"{fnbase1}_FLAIR.nii.gz"     # FLAIR.nii.gz
    SynthsegT1fn = f"{fnbase}_T1_synthseg_parc_robust.nii.gz"     # T1 SynthSeg s

    chns = [T1fn, CT1fn, T2fn, FLAIRfn]

    print(f"\npas: {sub}, ses: {ses}")
    for i, c in enumerate(chns):
        print(f" chn{i}: {c}")
        img = nib.load(c)
        print(f"    shape: {img.shape}, voxels: {img.header.get_zooms()}")
    print(f" SynthsegT1: {SynthsegT1fn}")
    img = nib.load(SynthsegT1fn)
    print(f"    shape: {img.shape}, voxels: {img.header.get_zooms()}")
```

In [ ]:

```
def get_shape_res2(base1, base2, exam):  # sub, ses):

    sub = exam[0:1]
    ses = exam[2:4]
    fnbase1 = f"{base1}/{exam}/{sub}_{ses}"
    fnbase2 = f"{base2}/{exam}/{sub}_{ses}"
    T1fn = f"{fnbase1}_T1.defaced.nii.gz"          # T1.nii.gz
    CT1fn = f"{fnbase1}_CT1.defaced.nii.gz"        # CT1.nii.gz
    T2fn = f"{fnbase1}_T2.defaced.nii.gz"          # T2.nii.gz
    FLAIRfn = f"{fnbase1}_FLAIR.defaced.nii.gz"    # FLAIR.nii.gz
    SynthsegT1fn = f"{fnbase2}_T1_synthseg_parc_robust.nii.gz"    # T1 SynthSeg

    chns = [T1fn, CT1fn, T2fn, FLAIRfn]

    print(f"\npas: {sub}, ses: {ses}")
    for i, c in enumerate(chns):
        print(f" chn{i}: {c}")
        img = nib.load(c)
        print(f"   shape: {img.shape}, voxels: {img.header.get_zooms()}")
    print(f" SynthsegT1: {SynthsegT1fn}")
    img = nib.load(SynthsegT1fn)
    print(f"   shape: {img.shape}, voxels: {img.header.get_zooms()}")
```

In [ ]:

```
exams_list = sorted([x for x in next(os.walk(nifti_dir))[1]])
exams_list
```

In [ ]:

```
for ex in exams_list[0:1]:
    get_shape_res2(nifti_dir, synthseg_dir, ex)
```

Get image matrices and voxel resolutions for each channel

In [ ]:

```
def get_shape_res(base, exam):  # sub, ses):

    sub = exam[0:1]
    ses = exam[2:4]
    fnbase = f"{base}/{exam}/{sub}_{ses}"
    T1fn = f"{fnbase}_T1.nii.gz"          # T1.nii.gz
    CT1fn = f"{fnbase}_CT1.nii.gz"        # CT1.nii.gz
    T2fn = f"{fnbase}_T2.nii.gz"          # T2.nii.gz
    FLAIRfn = f"{fnbase}_FLAIR.nii.gz"    # FLAIR.nii.gz
    SynthsegT1fn = f"{fnbase}_T1_synthseg_parc_robust.nii.gz"    # T1 SynthSeg s

    chns = [T1fn, CT1fn, T2fn, FLAIRfn]

    print(f"\npas: {sub}, ses: {ses}")
    for i, c in enumerate(chns):
        print(f" chn{i}: {c}")
        img = nib.load(c)
        print(f"   shape: {img.shape}, voxels: {img.header.get_zooms()}")
    print(f" SynthsegT1: {SynthsegT1fn}")
    img = nib.load(SynthsegT1fn)
    print(f"   shape: {img.shape}, voxels: {img.header.get_zooms()}")
```

Test case (e.g. `B_01`) used for each of the steps (as functions)

In [ ]:

```
base = test_hd_glio
results = test_hd_glio
#exams_list
```

In [ ]:

```
exam = exams_list[2:3][0]
sub = exam[0:1]
ses = exam[2:4]

x, y, z = 40, 20, 15  ## (generally obtained from HD-GLIO and nilearn's: plottir
x, y, z = -5, -31, 54
print(f"test case: {exam}, i.e. sub={sub}, ses={ses}")
```

In [ ]:

```
for exam in exams_list[2:3]:
    print(f"exam: {exam}")

    src_dir = f"{nifti_dir}/{exam}"     # Bortem_nifti_for_hdglio_pseudonym
    ss_dir = f"{synthseg_dir}/{exam}"   # Bortem_synthseg_pseudonym
    dst_dir = f"{results}/{exam}"       # Bortem_hdglio_pseudonym

    if not os.path.exists(dst_dir):
        os.makedirs(dst_dir)
        #print(dst_dir)

    for name in glob.glob(f"{src_dir}/*.nii.gz"):
        src_file = f'{name}'
        head, tail_deface = os.path.split(src_file)
        print(f"head: {head}")
        print(f"tail_deface: {tail_deface}")
        tail = tail_deface.replace('.defaced', '')  # Omit .defaced substring in
        dst_file = f"{dst_dir}/{tail}"
        print(src_file, ' to ', dst_file, '\n')
        shutil.copy(src_file,dst_file)

    # Copy corresponding SynthSeg file(s)
    for name in glob.glob(f"{ss_dir}/*T1_synthseg_parc_robust.nii.gz"):
        src_file = f'{name}'
        head, tail = os.path.split(src_file)
        dst_file = f"{dst_dir}/{tail}"
        print(src_file, ' to ', dst_file, '\n')
        shutil.copy(src_file,dst_file)
```

In [ ]:

```
%%time
## Get geometric info:
get_shape_res(results, exam)
```

Display the test case and the SynthSeg segmentation

In [ ]:

```
%%time
## Inspect template examination

fn_inp = f"{base}/{exam}/{sub}_{ses}_{template}.nii.gz"
ss_inp = f"{base}/{exam}/{sub}_{ses}_{template}_synthseg_parc_robust.nii.gz"
img = nib.load(fn_inp)
msk = nib.load(ss_inp)

fig, ax  = plt.subplots(1, 1, figsize=(15,6))
display = plotting.plot_anat(img, axes = ax, title=f"{sub}_{ses}: {template}",
                             cmap="gray", dim= -0.7, cut_coords = (x, y, z))
plt.show()
```

In [ ]:

```
fig, ax  = plt.subplots(1, 1, figsize=(15,6))
display = plotting.plot_anat(img, axes = ax, title=f"{sub}_{ses}: {template}_syr
                             cmap="gray", dim= -0.9, cut_coords = (x, y, z))
display.add_overlay(msk, cmap=plotting.cm.black_green)
plt.show()
```

Reslice the channels to the one with the highest resolution (T1) using FS's `mri_convert`

In [ ]:

```
def mriconvert_like_chn(fn_templ, fn_inp, fn_out):

    '''
    Freesurfer's mri_convert:
    mri_convert --like fn_t1 fn_t2 fn_t2_t1
    '''

    cmd = [
        MRICONVERT,
        '--like', fn_templ,
        fn_inp, fn_out]
    cmd_str = " ".join(cmd)

    # EXECUTE
    # os.system(cmd_str), or:
    output = subp.check_output(cmd)  # import subprocess as subp
    print(f'sub: {sub}')
    print(output,'\n')




def reslice_like(base, results, exam, chn, template):

    sub = exam[0:1]
    ses = exam[2:4]

    print(f"sub: {sub}")
    print(f"ses: {ses}")
    res_dir = f"{results}/{exam}"
    if not os.path.exists(res_dir):
        os.makedirs(res_dir)
    fn_inp = f"{base}/{exam}/{sub}_{ses}_{chn}.nii.gz"
    fn_templ = f"{base}/{exam}/{sub}_{ses}_{template}.nii.gz"
    fn_out = f"{base}/{exam}/{sub}_{ses}_{chn}_like_{template}.nii.gz"

    print(f"fn_inp: {fn_inp}\nfn_templ: {fn_templ}\nfn_out: {fn_out}")

    if chn == template:  # just copy and rename
        src = fn_inp
        dst = fn_out
        shutil.copy(src,dst)

        src_synthseg = f"{base}/{exam}/{sub}_{ses}_{chn}_synthseg_parc_robust.ni
        dst_synthseg = f"{base}/{exam}/{sub}_{ses}_{chn}_synthseg_parc_robust_li
        shutil.copy(src_synthseg,dst_synthseg)
    else:
        mriconvert_like_chn(fn_templ, fn_inp, fn_out)
```

In [ ]:

```
%time
# Reslice the other (non-template) sequences in the mpMRI

p_chns = ['T1', 'CT1', 'T2', 'FLAIR']
or i, ch in enumerate(mp_chns):
    print(f"{i}: {ch}")
    reslice_like(base, results, exam, ch, template)

# Inspect non-template channel
hn = 'T2'

ub = exam[0:1]
es = exam[2:4]

n_inp = f"{base}/{exam}/{sub}_{ses}_{chn}_like_{template}.nii.gz"

mg = nib.load(fn_inp)

ig, ax  = plt.subplots(1, 1, figsize=(15,6))
lotting.plot_anat(img, axes = ax, title=f"{sub}_{ses} - chn: {chn}", cmap="gray", di
lt.show()
```

Reorient the resliced channels and the template to standard space using FSL6's `fslreorient2std`

In [ ]:

```
def reorient(results, exam, chn, template):

    sub = exam[0:1]
    ses = exam[2:4]


    fn_inp = f"{results}/{exam}/{sub}_{ses}_{chn}_like_{template}.nii.gz"


    cmd = [
        FSLREORIENT2STD,
        fn_inp,
        '%s_reorient.nii.gz' % (fn_inp[:-7])]  # skip .nii.gz
    cmd_str = " ".join(cmd)

    # EXECUTE
    #os.system(cmd_str), or:
    output = subp.check_output(cmd)  # import subprocess as subp
    print(cmd_str,'\n')
```

In [ ]:

```
%%time
## Reorient the reslices channels and the template to standard  space using FSL6

for i, ch in enumerate(all_chns_synthseg):

    print(f"{i}: {ch}")
    reorient(results, exam, ch, template)


## Inspect template channel
chn = 'T1'
fn_inp = f"{base}/{exam}/{sub}_{ses}_{chn}_like_{template}.nii.gz"
img = nib.load(fn_inp)
fig, ax  = plt.subplots(1, 1, figsize=(15,6))
plotting.plot_anat(img, axes = ax, title=f"{sub}_{ses} - chn: {chn}", cmap="gray
plt.show()

## Inspect template channel
chn = 'CT1'
fn_inp = f"{base}/{exam}/{sub}_{ses}_{chn}_like_{template}.nii.gz"
img = nib.load(fn_inp)
fig, ax  = plt.subplots(1, 1, figsize=(15,6))
plotting.plot_anat(img, axes = ax, title=f"{sub}_{ses} - chn: {chn}", cmap="gray
plt.show()

## Inspect non-template channel
chn = 'FLAIR'
fn_inp = f"{base}/{exam}/{sub}_{ses}_{chn}_like_{template}.nii.gz"
img = nib.load(fn_inp)
fig, ax  = plt.subplots(1, 1, figsize=(15,6))
plotting.plot_anat(img, axes = ax, title=f"{sub}_{ses} - chn: {chn}", cmap="gray
plt.show()
```

Perform HD-BET skull stripping of a channel

e.g.

```
hd-bet -i CT1_reorient.nii.gz -o ct1_bet.nii.gz
```

In [ ]:

```
!which hd-bet
```

In [ ]:

```
!hd-bet -h
```

In [ ]:

```
def hdbet(chn_fn):
    '''
    Producing:
    <chn_fn>_bet_mask.nii.gz and _
    <chn_fn>_bet.nii.gz
    Note: GeForce RTX 3090 with CUDA capability sm_86 is not compatible with the
          The current PyTorch install supports CUDA capabilities sm_37 sm_50 sm_
          If you want to use the GeForce RTX 3090 GPU with PyTorch,
          please check the instructions at https://pytorch.org/get-started/local
    '''

    cmd = [
        HDBET,
        '-i', chn_fn,
        '-device',  '0'  # '1' is TITAN V  on medgpu1;   ('0' is RTX 3090)
    ]
    cmd_str = " ".join(cmd)

    # EXECUTE
    #os.system(cmd_str)
    output = subp.check_output(cmd)  # import subprocess as subp
    print('cmd_str = \n%s' % cmd_str)
    print(f'output:\n{output}\n')
```

In [ ]:

```
%%time
## Skull stripping of each channel with HD-BET
for i, ch in enumerate(all_chns):


    print(f'HD-BET {i}: {ch}')
    fn_inp = f"{results}/{exam}/{sub}_{ses}_{ch}_like_{template}_reorient.nii.gz


    hdbet(fn_inp)
```

In [ ]:

```
# Inspect HD-BET results

for i, chn in enumerate(all_chns):

    # sub = exam[0:1]
    # ses = exam[2:4]

    fn_inp = f"{results}/{exam}/{sub}_{ses}_{chn}_like_{template}_reorient_bet.n
    img = nib.load(fn_inp)
    fig, ax  = plt.subplots(1, 1, figsize=(15,6))
    plotting.plot_anat(img, axes = ax, title=f"HD-BET: {sub}_{ses} - chn: {chn}'
                       cmap="gray", dim= -0.7, cut_coords = (x, y, z))
    plt.show()
```

Register all skull-stripped sequences to the template (T1) channel using FSL's **`flirt`**, save matrix

flirt (https://fsl.fmrib.ox.ac.uk/fsl/fslwiki/FLIRT) is a fully automated robust and accurate tool for linear (affine) intra- and inter-modal brain image registration, here using 6 DOF and *spline* interplotation.
See also the FLIRT / UserGuide (https://fsl.fmrib.ox.ac.uk/fsl/fslwiki/FLIRT/UserGuide).

e.g.

```
flirt -in ct1_bet.nii.gz -out ct1_bet_reg.nii.gz -ref t1_bet.nii.gz -omat ct
1_to_t1.mat -interp spline -dof 6
```

In [ ]:

```python
def register2template(moving_chn, fixed_chn, template):


    cmd = [
        FLIRT,
        '-in',
        moving_chn,
        '-ref',
        fixed_chn,
        '-out',
        '%s_reg.nii.gz' % moving_chn[:-7],
        '-omat',
        '%s_to_%s.mat' % (moving_chn[:-7], template),
        '-dof', '6',
        '-interp', 'spline',
        '-verbose', '1'
    ]
    cmd_str = " ".join(cmd)

    # EXECUTE
    #os.system(cmd_str), or
    output = subp.check_output(cmd)  # import subprocess as subp
    print('cmd_str = \n%s' % cmd_str)
    print(f'output:\n{output}\n')

```

In [ ]:

```
def register2template_seg(moving_msk, fixed_chn, template):


    cmd = [
        FLIRT,
        '-in',
        moving_msk,
        '-ref',
        fixed_chn,
        '-out',
        '%s_reg.nii.gz' % moving_msk[:-7],
        '-omat',
        '%s_to_%s.mat' % (moving_msk[:-7], template),
        '-dof', '6',
        '-interp', 'nearestneighbour',
        '-verbose', '1'
    ]
    cmd_str = " ".join(cmd)

    # EXECUTE
    #os.system(cmd_str), or
    output = subp.check_output(cmd)  # import subprocess as subp
    print('cmd_str = \n%s' % cmd_str)
    print(f'output:\n{output}\n')

```

In [ ]:

```
%%time
## Register (affinely) all skull-stripped sequences to the template channel usi
for i, ch in enumerate(inp_chns):
    fn_fixed = f"{results}/{exam}/{sub}_{ses}_{template}_like_{template}_reorien
    fn_moving = f"{results}/{exam}/{sub}_{ses}_{ch}_like_{template}_reorient_bet
    #print(f'{i}: {ch} Fixed: {fn_fixed}')
    register2template(fn_moving, fn_fixed, template)

```

In [ ]:

```
%%time
## Register (affinely) synthseg mask to the template channel using FSL's `flirt

ch = 'T1_synthseg_parc_robust'
fn_fixed = f"{results}/{exam}/{sub}_{ses}_{template}_like_{template}_reorient_be
fn_moving = f"{results}/{exam}/{sub}_{ses}_{ch}_like_{template}_reorient_bet.nii
    #print(f'{i}: {ch} Fixed: {fn_fixed}')
# register2template_seg(fn_moving, fn_fixed, template)
```

In [ ]:

```
## Inspect

for i, chn in enumerate(all_chns[1::]):
    fn_omat = f"{results}/{exam}/{sub}_{ses}_{chn}_like_{template}_reorient_bet_
    omat_title = f"{chn}: {sub}_{ses}_{chn}_like_{template}_reorient_bet_to_{tem
    fn_inp = f"{results}/{exam}/{sub}_{ses}_{chn}_like_{template}_reorient_bet_r
    img = nib.load(fn_inp)
    fig, ax  = plt.subplots(1, 1, figsize=(15,6))
    plotting.plot_anat(img, axes = ax, title=omat_title, cmap="gray", dim= -0.7,
    plt.show()
```

In [ ]:

```
# Homogeneous coordinates
df_mat = pd.read_csv(fn_omat, delim_whitespace=True, header=None)
df_mat.columns = ['x', 'y', 'z', 'w']
df_mat
```

Apply the transformation matrices to the original images (pre hd-bet)

```
flirt -in CT1_reorient.nii.gz -out ct1_reg.nii.gz -ref t1_bet.nii.gz -applyx
fm -init ct1_to_t1.mat -interp spline
```

In [ ]:

```
def apply_omat2image_pre_hd_bet(moving_chn, ref_chn, template):

    cmd = [
        FLIRT,
        '-in',
        moving_chn,
        '-out',
        '%s_reg_ref_%s_bet.nii.gz' % (moving_chn[:-7], template),
        '-ref',
        ref_chn,
        '-applyxfm',
        '-init',
        '%s_bet_to_%s.mat' % (moving_chn[:-7], template),
        '-interp', 'spline',
        '-verbose', '1'
    ]
    cmd_str = " ".join(cmd)

    # EXECUTE
    #os.system(cmd_str), or
    output = subp.check_output(cmd)  # import subprocess as subp
    print('cmd_str = \n%s' % cmd_str)
    print(f'output:\n{output}\n')

```

In [ ]:

```
%%time
## Apply the transformation matrices to the original images (pre hd-bet)
for i, ch in enumerate(inp_chns):

    fn_moving = f"{results}/{exam}/{sub}_{ses}_{ch}_like_{template}_reorient.nii

    ref_chn = f"{results}/{exam}/{sub}_{ses}_{template}_like_{template}_reorient

    #print(f'{i}: {ch}')
    apply_omat2image_pre_hd_bet(fn_moving, ref_chn, template)

```

In [ ]:

```
## Inspect reorientation / coregistration to template

for i, chn in enumerate(all_chns):

    fn_inp = f"{base}/{exam}/{sub}_{ses}_{chn}_like_{template}_reorient.nii.gz"
    img = nib.load(fn_inp)
    tit = f"{chn}: {sub}_{ses}_{chn}_like_{template}_reorient.nii.gz"
    fig, ax  = plt.subplots(1, 1, figsize=(15,6))
    plotting.plot_anat(img, axes = ax, title=tit, cmap="gray", dim= -0.7, cut_co
    plt.show()
```

Reapply T1 brain mask using FSL's `fslmaths` (part of [FSLUTILS (https://fsl.fmrib.ox.ac.uk/fslcourse/lectures/practicals/intro3/index.html)](https://fsl.fmrib.ox.ac.uk/fslcourse/lectures/practicals/intro3/index.html)) to force non-brain voxels to be 0

This is important because HD-GLIO expects non-brain voxels to be 0.

```
-mas   : use (following image>0) to mask current image

#!fslmaths ./data/CT1_reorient_bet_reg.nii.gz -mas ./data/T1_reorient_bet_ma
sk.nii.gz ./data/CT1_reorient_bet_reg.nii.gz
```

e.g.

```
fslmaths ct1_reg.nii.gz -mas t1_bet_mask.nii.gz CT1_reorient_reg_bet.nii.gz
 & # t1_bet_mask.nii.gz was generated by hd-bet (see above)
```

In [ ]:

```
def reapply_mas_chn_brainmask(inp_chn, mas_chn, out_chn):


    cmd = [
        FSLMATHS,
        inp_chn,
        '-mas',
        mas_chn,
        out_chn,
    ]
    cmd_str = " ".join(cmd)

    # EXECUTE
    # os.system(cmd_str), or
    output = subp.check_output(cmd)  # import subprocess as subp
    print('cmd_str = \n%s' % cmd_str)
    print(f'output:\n{output}\n')

```

In [ ]:

```
%%time
## Reapply T1 brain mask (to force non-brain to be 0)
for i, ch in enumerate(inp_chns):

    fn_inp = f"{base}/{exam}/{sub}_{ses}_{ch}_like_{template}_reorient_reg_ref_{
    fn_mas = f"{base}/{exam}/{sub}_{ses}_{ch}_like_{template}_reorient_bet_mask.
    fn_out = f"{base}/{exam}/{sub}_{ses}_{ch}_like_{template}_reorient_bet_reg_r
    #print(f'{i}: {ch}')
    reapply_mas_chn_brainmask(fn_inp, fn_mas, fn_out)
```

In [ ]:

```
## Inspect template channel
chn = 'T1'

fn_inp = f"{base}/{exam}/{sub}_{ses}_{chn}_like_{template}_reorient_bet.nii.gz"
img = nib.load(fn_inp)

#OrthoSlicer3D(img.get_fdata(), affine= img.affine, title=chn).show()
tit = f"{sub}_{ses}_{chn}_like_{template}_reorient_bet.nii.gz"
fig, ax  = plt.subplots(1, 1, figsize=(18,8))
plotting.plot_anat(img, axes = ax, title=tit, cmap="gray", dim= -0.7, cut_coords
plt.show()
```

In [ ]:

```
## Inspect non-template channels
chns = ['CT1', 'T2', 'FLAIR']

fig, axes  = plt.subplots(3, 1, figsize=(18,24))
i=0
for ax in axes.flatten():
    chn = chns[i]
    fn_inp = f"{base}/{exam}/{sub}_{ses}_{chn}_like_{template}_reorient_bet_reg_

    img = nib.load(fn_inp)
    tit = fn_inp = f"{sub}_{ses}_{chn}_like_{template}_reorient_bet_reg_nonbrair
    plotting.plot_anat(img, axes = ax, title=tit, cmap="gray", dim= -0.7, cut_cc
    i=i+1

plt.show()
```

Perform HD-GLIO on the skull-stripped registered channels

After applying this example you would use T1_reorient.nii.gz, CT1_reorient_reg_bet.nii.gz, T2_reorient_reg_bet.nii.gz and FLAIR_reorient_reg_bet.nii.gz to proceed.

In [ ]:

```
!which hd_glio_predict
```

In [ ]:

```
!hd_glio_predict -h
```

In [ ]:

```
def hdglio_pred(fn_t1, fn_t1c, fn_t2, fn_flair, fn_out):


    cmd = [
        HDGLIO_PRED,
        '-t1', fn_t1,
        '-t1c', fn_t1c,
        '-t2', fn_t2,
        '-flair', fn_flair,
        '-o', fn_out,
    ]
    cmd_str = " ".join(cmd)

    # EXECUTE
    # os.system(cmd_str), or
    output = subp.check_output(cmd)  # import subprocess as subp
    print('cmd_str = \n%s' % cmd_str)
    print(f'output:\n{output}\n')

```

In [ ]:

```
%%time
## Run HD-GLIO predict

fn_t1 = f"{results}/{exam}/{sub}_{ses}_T1_like_{template}_reorient_bet.nii.gz"

fn_t1c = f"{results}/{exam}/{sub}_{ses}_CT1_like_{template}_reorient_bet_reg_nor

fn_t2 = f"{results}/{exam}/{sub}_{ses}_T2_like_{template}_reorient_bet_reg_nonbr

fn_flair = f"{results}/{exam}/{sub}_{ses}_FLAIR_like_{template}_reorient_bet_reg

fn_out = f"{results}/{exam}/{sub}_{ses}_4chn_like_{template}_hd_glio_predict.nii

hdglio_pred(fn_t1, fn_t1c, fn_t2, fn_flair, fn_out)

```

In [ ]:

```
## Inspect HD-GLIO segmentation results
chn = 'T1'

fn_inp = f"{results}/{exam}/{sub}_{ses}_4chn_like_{template}_hd_glio_predict.nii
img = nib.load(fn_inp)

tit = f"{sub}_{ses}_4chn_like_{template}_hd_glio_predict.nii.gz"
fig, ax  = plt.subplots(1, 1, figsize=(18,8))
plotting.plot_anat(img, axes = ax, title=tit, cmap="gray", dim= 0.5, cut_coords
plt.show()
```

In [ ]:

```
def display_channels_and_roi(T1, CT1, T2, FLAIR, roi, BG_IMG):
    channels = [T1, CT1, T2, FLAIR]
    img = nib.load(roi)
    data = img.get_fdata()
    values, counts = np.unique(data.astype(int), return_counts=True)
    unique = dict(zip(values,counts))
    coll = list(unique.items())
    unique = dict(coll[1:])
    for i, chn in enumerate(channels):
        if BG_IMG == 'T1' and i==0:
            fig, ax  = plt.subplots(1, 1, figsize=(15,6))
            plotting.plot_roi(roi, axes=ax, title=f'{os.path.split(roi)[1]} ({ur
                              bg_img=channels[i], alpha=0.8, dim=-0.9, cmap=cm)

        elif BG_IMG == 'CT1' and i==1:
            fig, ax  = plt.subplots(1, 1, figsize=(15,6))
            plotting.plot_roi(roi, axes=ax, title=f'{os.path.split(roi)[1]} ({ur
                              bg_img=channels[i], alpha=0.8, dim=-0.9, cmap=cm)

        elif BG_IMG == 'T2' and i==2:
            fig, ax  = plt.subplots(1, 1, figsize=(15,6))
            plotting.plot_roi(roi, axes=ax, title=f'{os.path.split(roi)[1]} ({ur
                              bg_img=channels[i], alpha=0.8, dim=-0.9, cmap=cm)

        elif BG_IMG == 'FLAIR' and i==3:
            fig, ax  = plt.subplots(1, 1, figsize=(15,6))
            plotting.plot_roi(roi, axes=ax, title=f'{os.path.split(roi)[1]} ({ur
                              bg_img=channels[i], alpha=0.8, dim=-0.9, cmap=cm)

        elif BG_IMG == 'Synthseg' and i==0:  # TO BE MODIFIED!
            fig, ax  = plt.subplots(1, 1, figsize=(15,6))
            plotting.plot_roi(roi, axes=ax, title=f'{os.path.split(roi)[1]} ({ur
                bg_img=nib.load(f'{results}/{exam}/{sub}_{ses}_4chn_like_T1_hd_g
                alpha=1.0, dim=-1.0, cmap=cm) #cmap='Paired')

```

In [ ]:

```
T1 = f'{results}/{exam}/{sub}_{ses}_T1_like_T1_reorient.nii.gz'
CT1 = f'{results}/{exam}/{sub}_{ses}_CT1_like_T1_reorient.nii.gz'
T2 = f'{results}/{exam}/{sub}_{ses}_T2_like_T1_reorient.nii.gz'
FLAIR = f'{results}/{exam}/{sub}_{ses}_FLAIR_like_T1_reorient.nii.gz'
roi = f'{results}/{exam}/{sub}_{ses}_4chn_like_T1_hd_glio_predict.nii.gz'
BG_IMG = 'FLAIR'

display_channels_and_roi(T1, CT1, T2, FLAIR, roi, BG_IMG)
```

In [ ]:

```
display_channels_and_roi(T1, CT1, T2, FLAIR, roi, 'T1')
```

In [ ]:

```
display_channels_and_roi(T1, CT1, T2, FLAIR, roi, 'CT1')
```

In [ ]:

```
display_channels_and_roi(T1, CT1, T2, FLAIR, roi, 'T2')
```

In [ ]:

```
BG_IMG = 'Synthseg'

display_channels_and_roi(T1, CT1, T2, FLAIR, roi, BG_IMG)
```

Calculate the CE (=1) and NE (=2) volumes

using `np.where()` and `np.prod(vox_size)`

In [ ]:

```
def hdglio_ce_ne_volumes(fn_segm):


    img = nib.load(fn_segm)
    vox_size = img.header.get_zooms()
    vox_vol = np.prod(vox_size)
    data = img.get_fdata()

    # Find voxel locations (CE and NE roi) corresponding to hd_glio_pred segment

    CE_roi = np.where(data == 1)
    X_CE = np.asarray(data[CE_roi[0][:],CE_roi[1][:],CE_roi[2][:]]) # The MRI ch
    CE_vol = (len(X_CE.flatten())*vox_vol).astype(int)

    NE_roi = np.where(data == 2)
    X_NE = np.asarray(data[NE_roi[0][:],NE_roi[1][:],NE_roi[2][:]]) # The MRI ch
    NE_vol = (len(X_NE.flatten())*vox_vol).astype(int)

    return vox_vol, CE_vol, NE_vol
```

In [ ]:

```
%%time
## Calculate the CE and NE volumes
#fn_segm = '%s/%s_%s/sub-%s_ses-%s_4chn_like_%s_hd_glio_predict.nii.gz' % (resul
fn_segm = f'{results}/{exam}/{sub}_{ses}_4chn_like_{template}_hd_glio_predict.ni

vox_vol, CE_vol, NE_vol = hdglio_ce_ne_volumes(fn_segm)
print(f'sub-{sub}_ses-{ses}: CE-volume={CE_vol}, NE-volume={NE_vol}, vox_vol={vo
```

In [ ]:

```
## Inspect HD-GLIO segmentation results
chn = 'T1'

fn_bg = f"{results}/{exam}/{sub}_{ses}_{chn}_like_{template}_reorient_bet.nii.gz
fn_segm = f"{results}/{exam}/{sub}_{ses}_4chn_like_{template}_hd_glio_predict.ni

bg_img = nib.load(fn_bg)
segm_img = nib.load(fn_segm)

tit = f"{sub}_{ses}_4chn_like_{template}_hd_glio_predict.nii.gz - CE_vol:{CE_vol

fig, ax  = plt.subplots(1, 1, figsize=(18,8))
plotting.plot_roi(segm_img, bg_img, axes = ax, title=tit, cmap=cm, dim= -0.9, cu
plt.show()
```

Start processing all subjects and all their examinations

In [ ]:

```
print(exams_list)
```

Copy all defaced exams to the results direcory

In [ ]:

```
%%time

nifti_dir   = f"{os.path.expanduser('~')}/prj/Bortem/Bortem_nifti_for_hdglio_pseu
synthseg_dir = f"{os.path.expanduser('~')}/prj/Bortem/Bortem_synthseg_pseudonym'
results = f"{os.path.expanduser('~')}/prj/Bortem/Bortem_hdglio_pseudonym"

for exam in exams_list:
    print(f"exam: {exam}")

    src_dir = f"{nifti_dir}/{exam}"     # Bortem_nifti_for_hdglio_pseudonym
    ss_dir = f"{synthseg_dir}/{exam}"   # Bortem_synthseg_pseudonym
    dst_dir = f"{results}/{exam}"       # Bortem_hdglio_pseudonym

    if not os.path.exists(dst_dir):
        os.makedirs(dst_dir)
        #print(dst_dir)

    for name in glob.glob(f"{src_dir}/*.nii.gz"):
        src_file = f'{name}'
        head, tail_deface = os.path.split(src_file)
        print(f"head: {head}")
        print(f"tail_deface: {tail_deface}")
        tail = tail_deface.replace('.defaced', '')  # Omit .defaced substring in
        dst_file = f"{dst_dir}/{tail}"
        print(src_file, ' to ', dst_file, '\n')
        shutil.copy(src_file,dst_file)

    # Copy corresponding SynthSeg file(s)
    for name in glob.glob(f"{ss_dir}/*T1_synthseg_parc_robust.nii.gz"):
        src_file = f'{name}'
        head, tail = os.path.split(src_file)
        dst_file = f"{dst_dir}/{tail}"
        print(src_file, ' to ', dst_file, '\n')
        shutil.copy(src_file,dst_file)
```

Make functions for extraction subject and list of session for that subject

In [ ]:

```
def get_list_of_subjects_from_exams_list(exams_list):
    s_list = set([x[0:1] for x in exams_list])
    subjects_list = sorted(list(s_list))
    return subjects_list

subjects_list = get_list_of_subjects_from_exams_list(exams_list)
subjects_list
```

In [ ]:

```
def get_list_of_sessions_for_a_subject(exams_list,sub):
    s_list = [x[0:1] for x in exams_list]
    occurrence = {item: s_list.count(item) for item in s_list}
    l = occurrence.get(sub)
    ses_list = []
    for i in range(l):
        ses_list.append(f"0{i+1}")

    return ses_list

ses = get_list_of_sessions_for_a_subject(exams_list, 'B')
ses
```

In [ ]:

```
ime

_chns = ['T1', 'CT1', 'T2', 'FLAIR']
_chns_synthseg = ['T1', 'CT1', 'T2', 'FLAIR', 'T1_synthseg_parc_robust']
plate = 'T1'

ılts = f"{os.path.expanduser('~')}/prj/Bortem/Bortem_hdglio_pseudonym"
e = results   # After the copying step from base to results above

y, z) = (0, 0, 0)   # Do not adjust center of ROI for each eaxamination

= pd.DataFrame(columns=['subj', 'ses', 'CE_vol', 'NE_vol'])


0


sub in subjects_list:


print(f'Subject:{sub}')


for ses in get_list_of_sessions_for_a_subject(exams_list, sub):

    print(f'  Session:{ses}')

    exam = f"{sub}_{ses}"


    ## Get geometric info

    get_shape_res(base, exam)




    ## Inspect template examination and the SynthSeg segmentation

    fn_inp = f"{base}/{exam}/{sub}_{ses}_{template}.nii.gz"
    ss_inp = f"{base}/{exam}/{sub}_{ses}_{template}_synthseg_parc_robust.nii.gz"
    img = nib.load(fn_inp)
    msk = nib.load(ss_inp)
    fig, ax  = plt.subplots(1, 1, figsize=(15,6))
    display = plotting.plot_anat(img, axes = ax, title=f"{sub}_{ses}: {template}",
                         cmap="gray", dim= -0.7, cut_coords = (x, y, z))
    plt.show()

    fig, ax  = plt.subplots(1, 1, figsize=(15,6))
    display = plotting.plot_anat(img, axes = ax, title=f"{sub}_{ses}: {template}_syn
                         cmap="gray", dim= -0.9, cut_coords = (x, y, z))
    display.add_overlay(msk, cmap=plotting.cm.black_green)
    plt.show()



    ## Reslice the channels to the one of highest resolution (T1) using FS's mri_con

    mp_chns = ['T1', 'CT1', 'T2', 'FLAIR']
    for i, ch in enumerate(mp_chns):
```

```
        reslice_like(base, results, exam, ch, template)



    ## Reorient the resliced channels and the template to standard space using FSL6

    for i, ch in enumerate(all_chns_synthseg):
        reorient(results, exam, ch, template)



    ## Skull stripping of each channel with HD-BET

    for i, ch in enumerate(all_chns):
        fn_inp = f"{results}/{exam}/{sub}_{ses}_{ch}_like_{template}_reorient.nii.gz
        hdbet(fn_inp)



    ## Register (affinely) all skull-stripped sequences to the template channel usin

    for i, ch in enumerate(inp_chns):
        fn_fixed = f"{results}/{exam}/{sub}_{ses}_{template}_like_{template}_reorien
        fn_moving = f"{results}/{exam}/{sub}_{ses}_{ch}_like_{template}_reorient_bet
        register2template(fn_moving, fn_fixed, template)




    ## Apply the transformation matrices to the original images (pre hd-bet)

    for i, ch in enumerate(inp_chns):
        fn_moving = f"{results}/{exam}/{sub}_{ses}_{ch}_like_{template}_reorient.nii
        ref_chn = f"{results}/{exam}/{sub}_{ses}_{template}_like_{template}_reorient
        apply_omat2image_pre_hd_bet(fn_moving, ref_chn, template)



    ## Reapply T1 brain mask using FSL's fslmaths (part of FSLUTILS) to force non-bı

    for i, ch in enumerate(inp_chns):
        fn_inp = f"{base}/{exam}/{sub}_{ses}_{ch}_like_{template}_reorient_reg_ref_{
        fn_mas = f"{base}/{exam}/{sub}_{ses}_{ch}_like_{template}_reorient_bet_mask.
        fn_out = f"{base}/{exam}/{sub}_{ses}_{ch}_like_{template}_reorient_bet_reg_r
        reapply_mas_chn_brainmask(fn_inp, fn_mas, fn_out)



    ## Perform HD-GLIO prediction on the skull-stripped registered channels

    fn_t1 = f"{results}/{exam}/{sub}_{ses}_T1_like_{template}_reorient_bet.nii.gz"

    fn_t1c = f"{results}/{exam}/{sub}_{ses}_CT1_like_{template}_reorient_bet_reg_nor

    fn_t2 = f"{results}/{exam}/{sub}_{ses}_T2_like_{template}_reorient_bet_reg_nonbr

    fn_flair = f"{results}/{exam}/{sub}_{ses}_FLAIR_like_{template}_reorient_bet_reg

    fn_out = f"{results}/{exam}/{sub}_{ses}_4chn_like_{template}_hd_glio_predict.nii

    hdglio_pred(fn_t1, fn_t1c, fn_t2, fn_flair, fn_out)
```

```
    ## Calculate the CE (=1) and NE (=2) volumes using np.where() and np.prod(vox_si

    fn_segm = f'{results}/{exam}/{sub}_{ses}_4chn_like_{template}_hd_glio_predict.ni
    vox_vol, CE_vol, NE_vol = hdglio_ce_ne_volumes(fn_segm)
    print(f"sub-{sub}_ses-{ses}: CE-volume={CE_vol}, NE-volume={NE_vol}, vox_vol={vo



    ## Add results to dataframe df

    df.loc[ii] = [sub, ses, CE_vol, NE_vol]

    ii = ii+1



Save to .csv file

:y = date.today().strftime("%Y%m%d")
:sv = f"{results}/bortem_hd_glio_ce_ne_volumes_{today}.csv"
:o_csv(fn_csv, index=False)
```

Timing

Dell Precision-7560: 29 examinations (13 subjects)

```
CPU times: user 4min 46s, sys: 7.53 s, total: 4min 53s
Wall time: 1h 50min 31s
```

In [ ]:

```
today = date.today().strftime("%Y%m%d")
```

In [ ]:

```
fn_csv = f"{results}/bortem_hd_glio_ce_ne_volumes_{today}.csv"
fn_csv
```

In [ ]:

```
df.to_csv(fn_csv, index=False)
```

In [ ]:

```
df
```

In [ ]:

```
df1 = pd.read_csv(f"{results}/bortem_hd_glio_ce_ne_volumes_2022xxyy.csv")
```

AD HOC visualization in Freesurfer's freeview

[illegible]

In [ ]:

```
subj = 'B'
sess = '01'
```

In [ ]:

```
%%bash -s "$subj" "$sess" "$home"

echo "subject: $1"
echo "session: $2"

FREESURFER_HOME=/usr/local/freesurfer/7.3.2; export FREESURFER_HOME
PATH=${FREESURFER_HOME}/bin:${PATH}; export PATH
SUBJECTS_DIR=/home/arvid/prj/Bortem/Bortem_hdglio_pseudonym; export SUBJECTS_DI
FSLDIR=/usr/local/fsl; export FSLDIR
PATH=${FSLDIR}/bin:${PATH}; export PATH
. ${FSLDIR}/etc/fslconf/fsl.sh
source ${FREESURFER_HOME}/SetUpFreeSurfer.sh

cd $SUBJECTS_DIR

freeview -v \
$1_$2/$1_$2_T1_like_T1_reorient.nii.gz \
$1_$2/$1_$2_T1_like_T1_reorient_bet.nii.gz \
$1_$2/$1_$2_CT1_like_T1_reorient_bet_reg_nonbrain_0.nii.gz \
$1_$2/$1_$2_T2_like_T1_reorient_bet_reg_nonbrain_0.nii.gz \
$1_$2/$1_$2_FLAIR_like_T1_reorient_bet_reg_nonbrain_0.nii.gz \
$1_$2/$1_$2_4chn_like_T1_hd_glio_predict.nii.gz:colormap=Jet:opacity=0.7 \
$1_$2/$1_$2_T1_synthseg_parc_robust_like_T1_reorient.nii.gz:colormap=lut:opacity
-ras -5 -10 35
```

Visual inspection (TO DO)

In [ ]:

```
from IPython.display import Image
#Image(filename='./assets/hd-glio_B_01.png', width=900)
```

In [ ]:

```

```
